# Single-cell imaging reveals a key role of Bck2 in yeast cell size adaptation to nutrient challenges

**DOI:** 10.1083/jcb.202410020

**Published:** 2026-10-09

**Authors:** Yagya Chadha, Igor V. Kukhtevich, Timon Stegmaier, Francesco Padovani, Robert Schneider, Kurt M. Schmoller

**Affiliations:** 1 Institute of Functional Epigenetics, Molecular Targets and Therapeutics Center, Helmholtz Zentrum München, Neuherberg, Germany

## Abstract

Cell size is tightly controlled to optimize cell function. In *Saccharomyces cerevisiae,* multiple regulators of cell size in steady-state conditions have been identified, such as the G1/S activators Cln3 and Bck2 and the inhibitor Whi5. Individual deletions of these regulators alter the mean cell volumes. However, size homeostasis remains largely intact. Here, we show that although the roles of Bck2 and Cln3 for cell size regulation appear largely redundant in steady state, a switch from fermentable to nonfermentable growth media reveals a unique role for Bck2 in cell size adaptation. We use live-cell microscopy and machine learning–assisted image analysis to track single cells and their progeny through the nutrient switch. We find that after the switch, *bck2Δ* cells experience longer cell cycle arrests and more arrest-associated enlargement than wild-type, *whi5Δ*, or *cln3Δ* cells, indicating that Bck2 becomes the critical G1/S activator in changing nutrients. Our work demonstrates that studying size regulation during nutrient shifts can resolve apparent redundancies observed in steady state.

## Teaser

Cells regulate their size according to nutrient conditions. Here, Chadha et al. use live-cell imaging and machine learning–assisted image analysis to follow budding yeast cell lineages as they adapt their size in response to a nutrient shift. They show that size regulators that seem redundant in steady state can have specific roles for cell size adaptation.

## Introduction

The size of a cell is a critical regulator of its function, and misregulation of cell size is associated with various disease states (Chadha et al., 2024). While the underlying causal relationships are often unclear, recent studies have revealed that increased cell size promotes cellular senescence and contributes to aging (Lengefeld et al., 2021; Manohar and Neurohr, 2024; Neurohr et al., 2019). It is therefore crucial that cell size is tightly controlled and adapted according to external and internal parameters, such as dynamic environments and developmental changes. Size homeostasis, i.e., the maintenance of narrow cell size distributions, is attributed to the cell’s ability to measure its size and coordinate cell cycle progression and cell growth with cell size (Cadart et al., 2018; Ginzberg et al., 2018; Soifer et al., 2016). For example, one common size-sensing strategy that has been reported for budding yeast (Schmoller et al., 2015), plant (D’Ario et al., 2021), and mammalian cells (Zatulovskiy et al., 2020; Zhang et al., 2024) is the cell growth–based concentration decrease of a cell cycle inhibitor.

However, while such specific size control mechanisms have been identified, there is growing evidence that eukaryotic size homeostasis is a product of multiple size control strategies (Garmendia-Torres et al., 2018; Miller et al., 2023). In fission yeast, for example, diverse mechanisms integrate cell surface area, cell volume, and time information into cell size control (Miller et al., 2023). Similarly, in budding yeast, the concentration of the size-sensing protein Whi5 alone accounts for the size-dependent likelihood of commitment to the G1/S transition in a *bck2Δ* strain (Schmoller et al., 2015), but multiple other activators and inhibitors of the same cell cycle transition have been identified (Chen et al., 2020; Soifer and Barkai, 2014). Accordingly, disruption of the Whi5-based mechanism only leads to a partial loss of size control (Chen et al., 2020; Barber et al., 2020; Swaffer et al., 2021).

**Figure 1.**
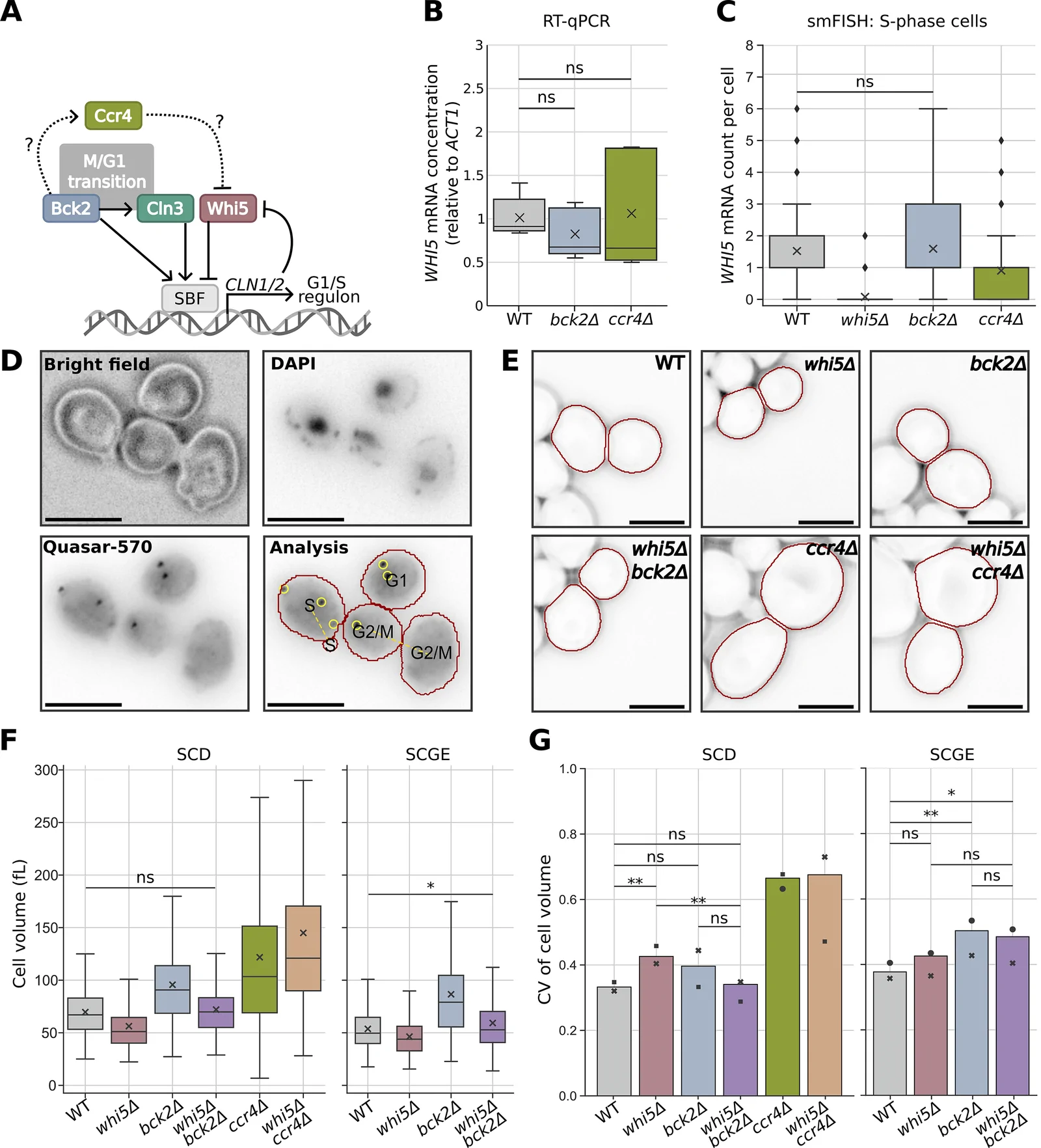
**Bck2 acts independently of Whi5. *whi5Δbck2Δ* has WT size and more efficient size homeostasis than *whi5Δ* and *bck2Δ* in glucose medium. (A)** Schematic illustration of key regulators of cell cycle progression and cell size at the budding yeast G1/S transition. **(B)** mRNA concentration of *WHI5* relative to *ACT1* in cells grown in SCD, as determined from five independent RT-qPCR experiments. **(C)***WHI5* mRNA count per cell from S-phase cells pooled from two independent smFISH experiments performed in SCD (n_WT_ = 172, n_*whi5Δ*_ = 182, n_*bck2Δ*_ = 211, n_*ccr4Δ*_ = 83). **(D)** Representative WT smFISH images: phase contrast; nuclear DNA stained with DAPI; *WHI5* mRNA stained with Quasar-570–labeled smFISH probes; the spots were detected using a custom routine in Python in yellow and cell contours in red. Dashed yellow lines connect mother–bud pairs. Cell cycle stage is annotated in black. Scale bars represent 5 µm. **(E)** Phase-contrast images from steady-state live-cell microscopy in SCD. Representative daughter cells (first-generation cells) from different strains are shown just before division and highlighted with red contours. Scale bars represent 5 µm. **(F)** Cell volume distributions obtained from steady-state live-cell microscopy in SCD and SCGE. For each strain, all living cells present in the last frame of each imaging position of two independent experiments were pooled. Cell numbers (*n*) for SCD: n_WT_ = 382, n_*whi5Δ*_ = 370, n_*bck2Δ*_ = 376, n_*whi5Δbck2Δ*_ = 463, n_*ccr4Δ*_ = 241, n_*whi5Δccr4Δ*_ = 280. Cell numbers for SCGE: n_WT_ = 533, n_*whi5Δ*_ = 370, n_*bck2Δ*_ = 429, n_*whi5Δbck2Δ*_ = 409. *ccr4Δ* and *whi5Δccr4Δ* cells growing in SCGE were excluded from analysis due to poor growth and high death rate. **(G)** Same dataset was used for calculating the CV of cell volume, as a measure of size homeostasis efficiency. •, ■, and **x** symbols are used to show the CVs of cell volume calculated from independent experimental replicates. For G, statistical analyses were performed by comparing overlaps between 10,000 bootstrap samples, explained in detail in the Materials and methods. Statistical comparisons were performed using two-sided Mann–Whitney U tests. Each strain was compared with WT and, where applicable, with *whi5Δbck2Δ*. For F and G, comparisons were made across both medium conditions separately (P < 0.001: not indicated for clarity; **: P < 0.01; *: P < 0.05; ns: not significant, P ≥ 0.05). For boxplots in B, C, and F, box limits show the IQR, whiskers extend to the most extreme data points within 1.5× IQR, the median is shown as a line, and the mean is shown as a cross. WT, wild type; IQR, interquartile range.

In budding yeast, size control through size-dependent cell cycle progression occurs mainly at the G1/S transition, and dilution by cell growth of the G1/S inhibitor Whi5 is one of the major underlying mechanisms (Schmoller et al., 2015; Qu et al., 2019). The G1/S transition is preceded by the commitment point *Start*, which is largely irreversible due to an underlying positive feedback loop controlling the G1/S transcription factors SBF and MBF (Fig. 1 A, Irvali et al., 2023; Skotheim et al., 2008). SBF is inhibited by Whi5 and activated by Cln3 and Bck2 (Bastajian et al., 2013; Costanzo et al., 2004; de Bruin et al., 2004; Kõivomägi et al., 2021). Once the positive feedback loop is triggered, Cln1 and Cln2 are expressed and, in complex with Cdk1, phosphorylate Whi5 to ensure the irreversibility of *Start*. All cells are born with roughly equal amounts of Whi5, and it is diluted by cell growth during G1 (Schmoller et al., 2015; Schmoller et al., 2022; Swaffer et al., 2021; Qu et al., 2019; Sommer et al., 2021; Xiao et al., 2024), whereas Cln3 and Bck2 concentrations remain mostly constant as G1 progresses (Schmoller et al., 2015; Chen et al., 2020). This widening imbalance between SBF activator and inhibitor concentrations is believed to incorporate cell size information into the cell cycle progression decision (Schmoller et al., 2015; Chen et al., 2020) and ultimately trigger the positive feedback loop.

Cln3 and Bck2 are both activators of the G1/S transition. Bck2 activates the G1/S transition in parallel to Cln3 by binding transcription factors Mcm1 and SBF at the *CLN2* promoter (Bastajian et al., 2013; Ferrezuelo et al., 2009). Additionally, it promotes the expression of Cln3 and the SBF subunit Swi4 at the M/G1 transition, and of the cyclin Clb2 at the G2/M transition (Bastajian et al., 2013). Deleting either Bck2 or Cln3 leads to an increased cell volume (Costanzo et al., 2004; Wijnen and Futcher, 1999), and the double deletion causes synthetic lethality (Epstein and Cross, 1994). Despite the changing average cell volumes, size homeostasis, assessed by the coefficient of variation (CV) of cell volume of a population, is surprisingly robust to single deletions of Cln3, Bck2, and Whi5, as well as other *Start* regulators (Chen et al., 2020). While such robustness could be an explanation for why cells evolved multiple redundant *Start* regulators, an alternative explanation is that the apparently redundant regulators fulfill unique roles under specific conditions that have not yet been identified.

Most size control studies so far have been conducted under steady-state conditions, i.e., during exponential growth in constant environments. Outside laboratory conditions, however, microorganisms frequently encounter rapid changes in the nutritional quality of the environment, which can lead to immediate effects on cellular decision making at the G1/S transition (Irvali et al., 2023). Moreover, nutrient availability is known to be a major regulator of cell size, with richer nutrients leading to larger cell sizes (Qu et al., 2019; Johnston et al., 1977; Leitao and Kellogg, 2017; Talavera et al., 2024). Changing nutrient conditions therefore require dynamic adaptation of cell size, and cell size regulators that appear redundant in constant growth conditions may have specific functions during this size adaptation. Indeed, previous findings point toward specific roles of Cln3 and Bck2 during nutrient changes: the Cln3 protein is very unstable with a half-life on the order of minutes (Tyers et al., 1992), and has therefore been proposed to act as a reporter for global cellular protein synthesis (Sommer et al., 2021; Tyers et al., 1992). Consistent with such a role, both the Cln3 protein and mRNA amounts are sensitive to nutrient availability (Hall, 1998; Parviz and Heideman, 1998), and a switch from a rich to a poor carbon environment is followed by an immediate Cln3 depletion (Sommer et al., 2021). Bck2, on the other hand, is not as well studied as Whi5 or Cln3, but has been shown to interact with multiple proteins with known roles in nutrient-sensing pathways (Bastajian et al., 2013). It has been proposed to be a link between the nutrient status of the environment and passage through the M/G1 transition (Bastajian et al., 2013). However, how Bck2 affects the dynamic adaptation of cell size to changing nutrient conditions is not clear.

Here, we use the budding yeast *Saccharomyces cerevisiae* as a model to investigate specific functions of apparently redundant cell size regulators during dynamic cell size adaptation to environmental changes. With microfluidics-based live-cell microscopy and state-of-the-art deep learning–based image analysis, we follow the fates of single cells and their complete lineages for multiple generations before and after a nutrient switch from fermentable to nonfermentable carbon sources. By following individual cells, we gain insights into the heterogeneity of cell size adaptation and find that the cell cycle state of a cell at the time of the nutrient shift plays a crucial role for the size adaptation of its offspring. Moreover, through analysis of deletion mutants, we uncover a unique role for Bck2. We find that after the nutrient switch, size homeostasis is temporarily disrupted, and this disruption is stronger in *bck2Δ* mutants, which show longer cell cycle arrests. Based on our findings, we propose that Bck2 acts as an essential G1/S transition activator for the period of time following a nutrient switch when Cln3 is depleted. More generally, our work suggests that the apparent redundancy of cell size regulators observed across eukaryotes in steady-state conditions may point to specific roles during dynamic cell size adaptation.

## Results

### Bck2 acts independently of Whi5

**Figure S1.**
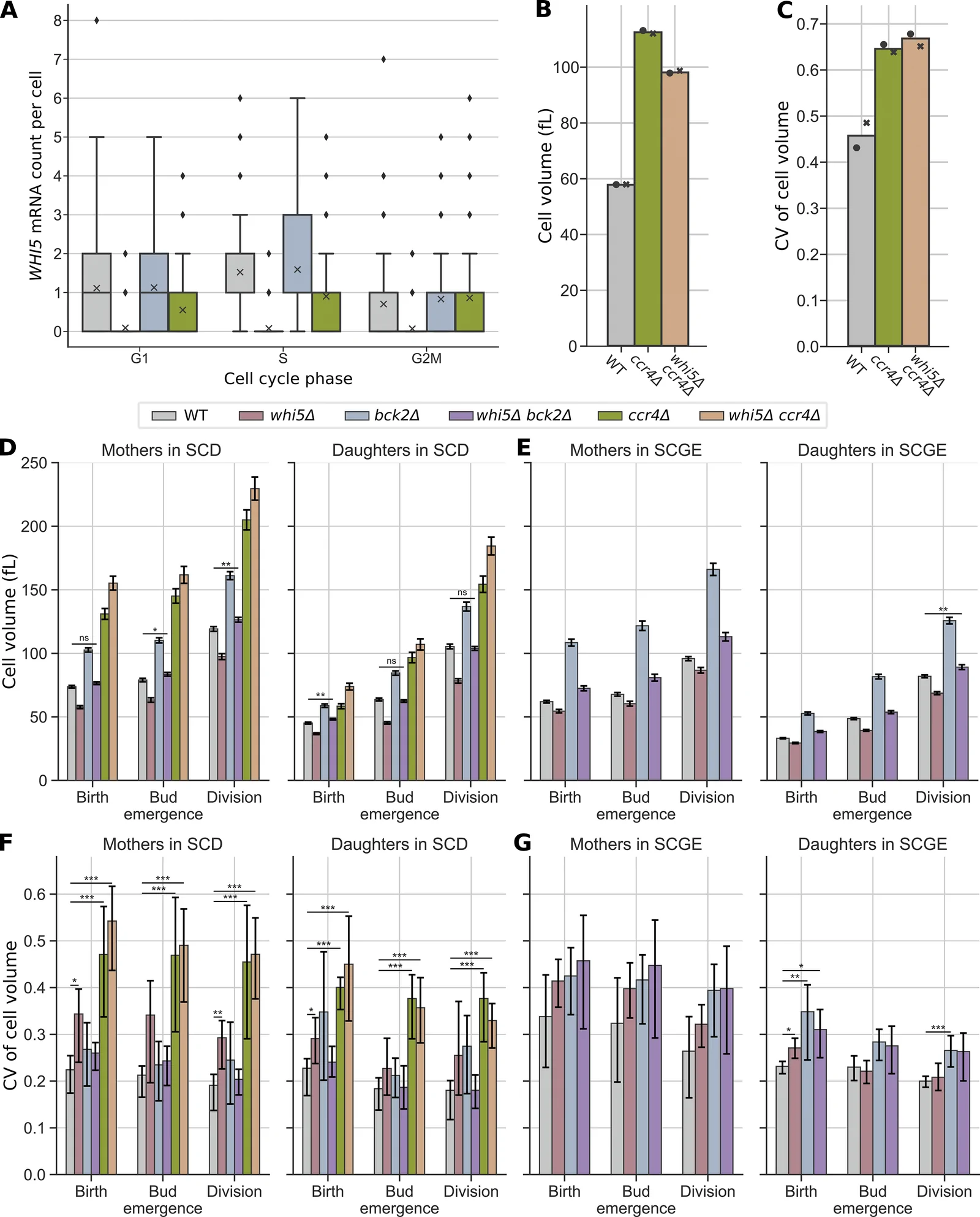
**Analysis of steady-state experiments. (A)** *WHI5* mRNA count per cell for cells in different cell cycle stages. Data are pooled from two independent smFISH experiments performed in SCD. G1 phase: n_WT_ = 98, n_*whi5Δ*_ = 121, n_*bck2Δ*_ = 196, n_*ccr4Δ*_ = 127. S phase: n_WT_ = 172, n_*whi5Δ*_ = 182, n_*bck2Δ*_ = 211, n_*ccr4Δ*_ = 83. G2/M phase: n_WT_ = 125, n_*whi5Δ*_ = 143, n_*bck2Δ*_ = 178, n_*ccr4Δ*_ = 130. Box limits show the IQR, whiskers extend to the most extreme data points within 1.5× IQR, the median is shown as a line, and the mean is shown as a cross. Outliers are additionally shown as diamonds. **(B and C)** Coulter counter measurements of cell volume (B) and CV of cell volume (C) are plotted for cells growing in SCD. Data are pooled from two independent experiments (• and **x** symbols). **(D and E)** Cell volume at birth, bud emergence, and division is plotted for daughter cells and mother cells growing in SCD (D) and SCGE (E). Two-sided Mann–Whitney U tests were used to statistically analyze each strain compared with WT (P < 0.001 not shown; **: P < 0.01; *: P < 0.05; ns: not significant, P ≥0.05). **(F and G)** CV of cell volume at birth, bud emergence, and division is plotted for daughter and mother cells growing in SCD (F) and SCGE (G). Statistical analyses were performed by comparing overlaps between 10,000 bootstrap samples, explained in detail in the Materials and methods. Error bars show 95th percentile confidence intervals. Significance was tested as explained in the Materials and methods, comparing each strain with WT (***: P < 0.001; **: P < 0.01; *: P < 0.05; not significant [P ≥ 0.05], not shown). WT, wild type; IQR, interquartile range.

Bck2 is mostly thought to promote the G1/S transition independently of Cln3 and Whi5 (Bastajian et al., 2013; Ferrezuelo et al., 2009). However, Manukyan et al. (2008) proposed that Bck2 stimulates the cytoplasmic deadenylase Ccr4, promoting degradation of *WHI5* mRNA (Fig. 1 A). Specifically, they found that *WHI5* mRNA was more stable in the absence of Ccr4 in a *GALpr*-*WHI5* strain after switching from galactose to glucose medium. Thus, as a first step to better understand the apparent redundancies of Bck2 and Cln3 for budding yeast G1/S size control, we decided to test this proposed interaction between Bck2, Ccr4, and Whi5 under steady-state conditions and for endogenous Whi5. We used RT–quantitative PCR (qPCR) (Fig. 1 B) and single-molecule fluorescence in situ hybridization (smFISH) (Fig. 1, C and D; and Fig. S1 A) to measure the effect of deleting *BCK2* or *CCR4* on *WHI5* transcript abundance for cells growing on glucose medium (synthetic complete medium with 2% glucose [SCD]), but did not see a significant increase. Next, to gain further insights into the genetic interaction between *CCR4* and *WHI5*, we performed time-lapse live-cell microscopy experiments (Fig. 1, E–G) and Coulter counter measurements (Fig. S1, B and C) of cell volume for single and double deletion strains. We found that *whi5Δ* cells were smaller, and *ccr4Δ* cells were much larger than wild-type cells. However, *whi5Δccr4Δ* did not rescue the large-cell phenotype of *ccr4Δ*. Taken together, our findings are not consistent with Ccr4 impacting cell size through destabilizing Whi5 mRNA. Instead, our data show that Whi5 and Ccr4 affect cell size via independent pathways.

We also performed time-lapse microscopy experiments for *WHI5* and *BCK2* single and double mutants growing on glucose medium (SCD), as well as glycerol–ethanol medium (synthetic complete medium with 2% glycerol and 1% ethanol [SCGE]). Consistent with previous studies (Chen et al., 2020; Costanzo et al., 2004; de Bruin et al., 2004; Di Como et al., 1995), *whi5Δ* and *bck2Δ* led to a significant decrease and increase in cell volume, respectively, and the volume of *whi5Δbck2Δ* cells was strikingly similar to that of wild type in both growth media (Fig. 1 F and Fig. S1, D–G). Thus, the effect of Bck2 and Whi5 on cell size appears to be additive, indicating that Whi5 is not downstream of Bck2 and that they affect cell size via independent paths.

### *whi5Δbck2Δ* has wild-type size and more efficient size homeostasis than *whi5Δ* and *bck2Δ* in glucose medium

After analyzing mean cell volumes, we next asked how size control is affected in each of the mutants. As a measure for size homeostasis efficiency, which is comparable between differently sized strains, we chose the CV of cell volume, i.e., the mean-normalized standard deviation of the cell volume distribution. Consistent with their roles in G1/S size control, we found that deletion of either *WHI5* or *BCK2* leads to an increased CV of cell volume, both for cells growing on SCD or SCGE (Fig. 1 G; and Fig. S1, F and G). We expected that deletion of both genes would lead to a further reduced size homeostasis efficiency. Surprisingly, the CV of cell volume of *whi5Δbck2Δ* cells was not higher than that of the single deletion mutants (Fig. 1 G). For cells growing in SCD, *whi5Δbck2Δ* even showed a significantly decreased CV compared with both Δ*whi5* and Δ*bck2* (Fig. 1 G), similar to wild-type cells. Thus, we found that size homeostasis as evaluated by the CV of the cell volume across a steady-state population was surprisingly robust to the double deletion of *WHI5* and *BCK2*, in particular for cells grown in SCD. In contrast, deletion of *CCR4* led to a dramatically increased CV of cell volume for cells grown on glucose, which was not rescued by an additional deletion of *WHI5* (Fig. 1 G). This indicates a rare occasion of a drastically weakened size homeostasis mechanism. However, given that *Δccr4* cells grow very poorly, especially in SCGE, it is unlikely that this lower size homeostasis is a result of disrupted G1/S size control alone.

### The increase in CV of cell volume observed after a nutrient switch is stronger in *whi5Δbck2Δ* than wild type

**Figure 2.**
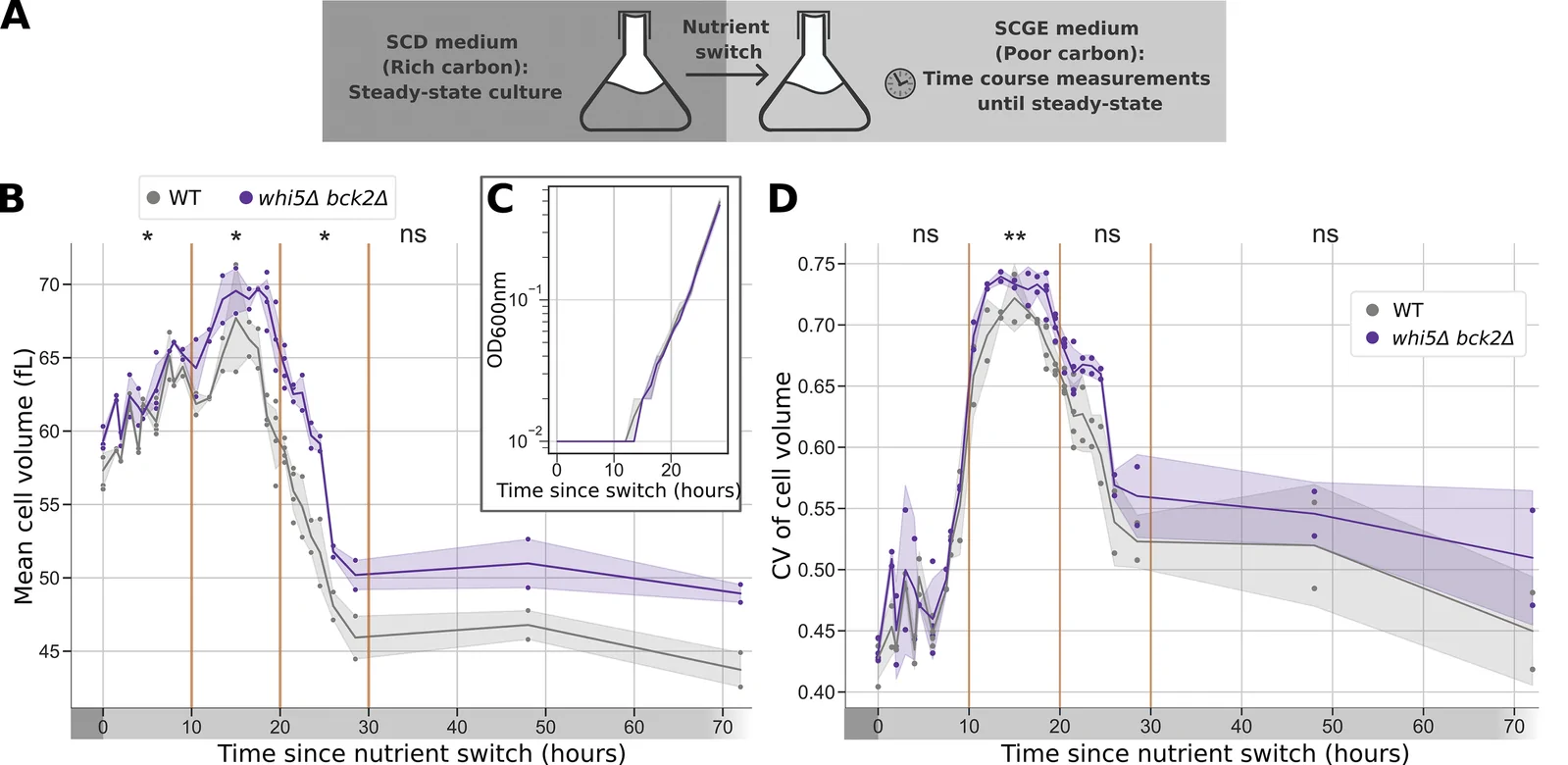
**Increase in CV of cell volume observed after a nutrient switch is stronger in *whi5Δbck2Δ* than wild-type cells. (A)** Illustration of the experiment design for the bulk nutrient switch experiment. Steady-state cells growing in SCD were washed and inoculated into SCGE, and a time course of OD_600nm_ and Coulter counter measurements were performed until cells reached steady state in SCGE. For the measurements taken 48 and 72 h after the nutrient switch, appropriate dilutions were made when necessary to maintain the OD_600nm_ of the cultures between 0.1 and 1. **(B and C)** Mean cell volume and OD_600nm_ (C, inset) are plotted against time since the nutrient switch. Shaded areas show 95% confidence intervals. Data for each time point are based on at least two experiments. **(D)** CV of cell volume is plotted against time since the nutrient switch. Shaded areas show 95% confidence intervals. For statistical analysis in B and D, the time course was divided into three 10-h windows (orange lines) and the remaining two time points were grouped together and analyzed with a mixed ANOVA test (see Materials and methods for details) (**: P < 0.01; *: P < 0.05; ns: not significant, P ≥ 0.05).

Prompted by the fact that the absence of Whi5 and Bck2, two major cell size regulators acting at the G1/S transition, does not have a stronger size homeostasis phenotype, even though deletion of each protein individually leads to altered cell volume and weakened size control, we speculated that double deletion of *WHI5* and *BCK2* may impair cell size adaptation upon changing nutrient conditions. To investigate this, we performed a bulk nutrient switch experiment, shifting cells from SCD to SCGE (Fig. 2 A). Specifically, steady-state populations of cells growing in SCD were washed and inoculated into SCGE (Fig. 2 A) at a starting OD_600nm_ of 0.01. We then performed a time course of OD_600nm_ and Coulter counter measurements of cell volume from the time of the nutrient switch up until a new steady state in the glycerol–ethanol medium was reached (Fig. 2, B–D). The OD_600nm_ was maintained between 0.1 and 1 through appropriate dilutions. For the first 15 h after the nutrient switch, both mean cell volume (Fig. 2 B) and CV of cell volume (Fig. 2 D) increased. Considering the direction of the switch was from a rich medium to a poorer medium, ultimately leading to a smaller mean cell volume after switch, the initial increase in cell volume was unexpected.

This initial increase in mean cell volume and CV of cell volume was not accompanied by notable cell proliferation. Only about 15 h after the switch, OD_600nm_ started to increase again, and at the same time, mean cell volume and CV of cell volume started to decrease (Fig. 2, B and D). Interestingly, during the time of maximal cell volume around 10–20 h after switch, *whi5Δbck2Δ* cells showed significant differences compared with wild type. We found that the CV of cell volume of *whi5Δbck2Δ* was significantly higher than that of wild type between 10 and 20 h after switch (Fig. 2 D). After 20 h, while the CV of *whi5Δbck2Δ* still appeared to be slightly higher than that of wild type for the remainder of the experiment, the difference was not statistically significant. Thus, both wild-type and *whi5Δbck2Δ *cells showed an increase in cell volume and CV of cell volume after switch, but *whi5Δbck2Δ* had lower size homeostasis efficiency than wild-type cells. This suggests that the simultaneous loss of both Whi5 and Bck2 does affect size homeostasis, but this effect is more prominent under changing nutrient conditions.

### Analysis strategy for live-cell microscopy coupled to nutrient switch

**Figure 3.**
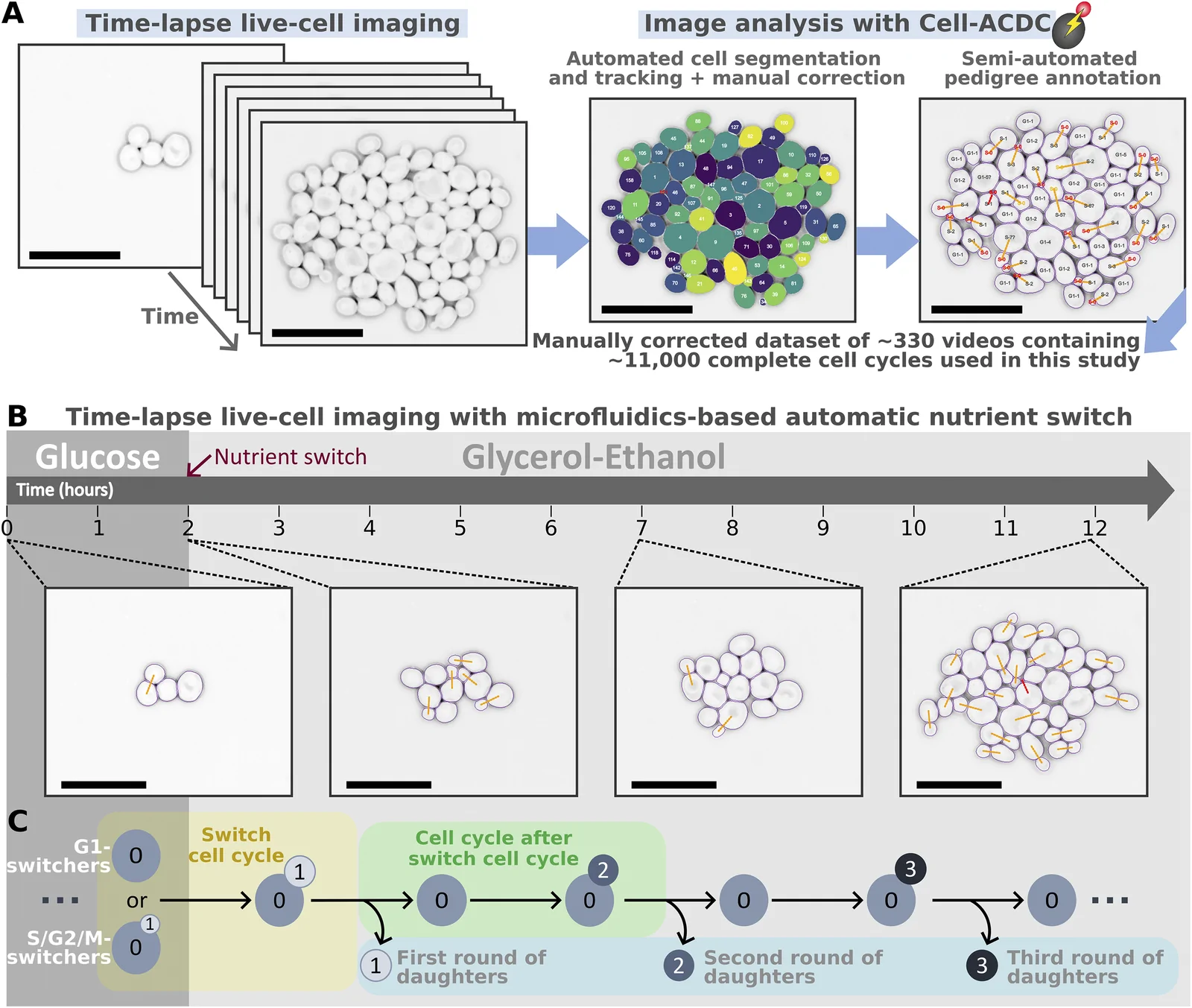
**Analysis strategy for live-cell microscopy of the adaptation to the nutrient switch. (A)** Live-cell imaging analysis pipeline for steady-state and nutrient switch experiments. Cell cycle stage is classified as G1 (unbudded cells) or S/G2/M (budded cells). **(B)** Representative images of WT cells in the nutrient switch live-cell microscopy experiment. **(C)** Cell categories of interest in the downstream analysis of the nutrient switch live-cell microscopy dataset. Scale bars represent 20 µm. WT, wild type.

To better understand why both strains underwent an increase in cell volume and CV of cell volume after the switch, and why *whi5Δbck2Δ* cells had a higher CV of cell volume, we performed live single-cell microscopy of cells experiencing the nutrient switch. We grew steady-state cultures of wild type, *whi5Δ*, *bck2Δ*, and *whi5Δbck2Δ* in SCD and transferred them to a custom microfluidics device (Kukhtevich et al., 2022) in a time-lapse microscopy setup (Fig. 3 A). We imaged the cells at 3-min intervals as they grew in SCD for 2 h and then automatically switched the medium to SCGE for the next 25 h. The videos of cell growth generated from this experiment were analyzed using the Cell-ACDC pipeline (Padovani et al., 2022; Padovani et al., 2025) and YeaZ (Dietler et al., 2020) for cell segmentation (Fig. 3 A). The cell masks and tracking were then manually corrected, and pedigrees were assigned in a semiautomated fashion in the Cell-ACDC GUI. Based on phase-contrast images, cells were classified as G1 (unbudded) or S/G2/M (budded) cells. This resulted in a fully annotated and manually corrected dataset of several thousand complete cell cycles (Fig. 3 A).

**Figure S2.**
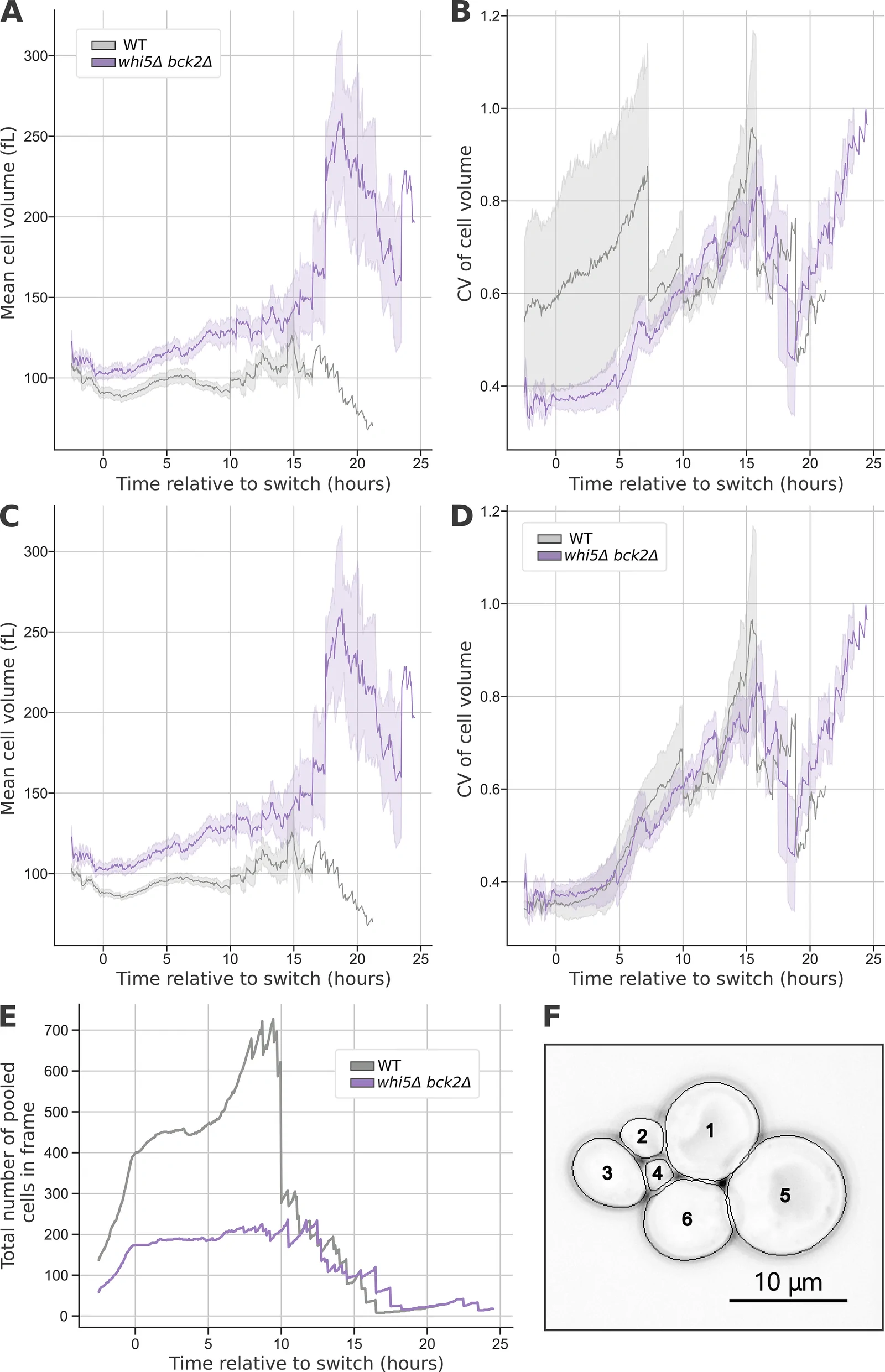
**Average behavior of a cell population, as calculated from single-cell measurements during the nutrient switch, is robust to outliers. However, measures of variability are sensitive to them. (A and B)** Mean cell volume (fL) and (B) CV of cell volume are plotted against time relative to the nutrient switch (hours). Bootstrapping with 1,000 iterations and replacement was used to obtain the standard error represented by shaded areas. The dataset for A and B includes all positions from three independent microscopy experiments. **(C and D)** In C and D, one outlier position with one very large cell at the beginning of the experiment is excluded from the dataset and the mean cell volume and CV of cell volume are recalculated. **(E)** Total number of pooled cells per frame is plotted against the time since the nutrient switch. **(F)** First frame of the outlier position excluded from the analysis in C and D is shown with black lines showing cell contours.

**Figure S3.**
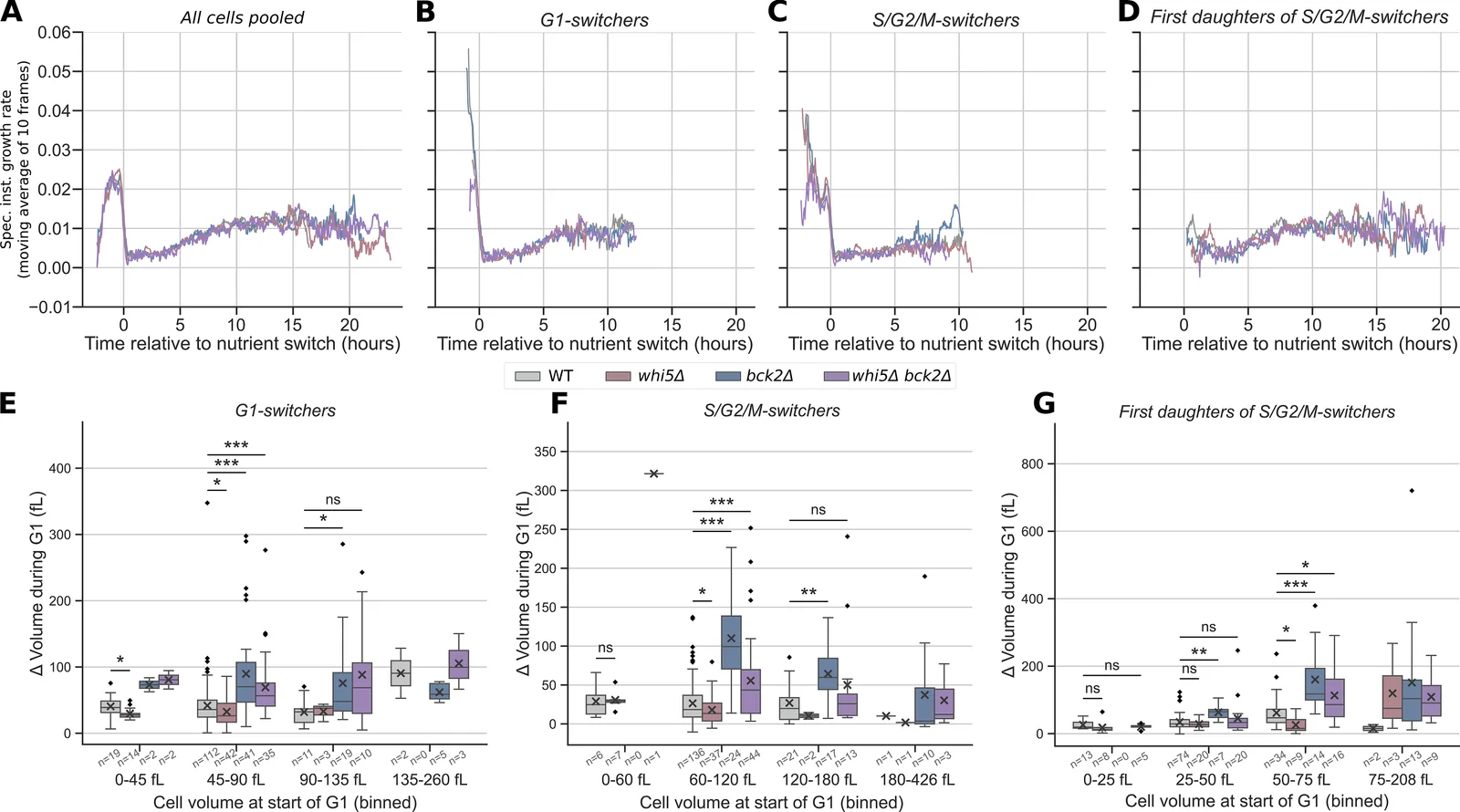
**Enlargement phenotype of *bck2Δ* cells is not explained by different durations of metabolic reprogramming or different starting cell volumes. (A–D)** Specific instantaneous growth rate after the nutrient shift is similar for wild-type and deletion mutants. The specific instantaneous growth rate per frame (1/3 min) is the change in volume between two frames normalized to cell volume. The moving average of the specific instantaneous growth rate over 10 frame (30-min) intervals is plotted against time relative to the nutrient switch. **(A)** All living cells from the nutrient switch experiment are pooled. **(B)** Only the switch cycles of G1-switchers are plotted. **(C)** Only the switch cycles of S/G2/M-switchers are plotted. **(D)** Only the first cell cycles of the first daughters of S/G2/M-switchers are plotted. **(E–G)** Change in cell volume during G1 is plotted for the *switch-cycle* of *G1-switchers* (E), the *cell-cycle-after-switch-cell-cycle* of *S/G2/M-switchers* (F), and the first G1 of *the first round of daughters of S/G2/M-switchers* (G). The x axis is the binned cell volume at the start of G1. Data shown correspond to Figs. 4 and 5. Significance was tested with two-sided Mann–Whitney U tests comparing the strains for each bin (***: P < 0.001; **: P < 0.01; *: P < 0.05; ns: not significant, P ≥ 0.05). No statistics are shown in case of a too low sample size. For boxplots in E–G, box limits show the IQR, whiskers extend to the most extreme data points within 1.5× IQR, the median is shown as a line, and the mean is shown as a cross. Outliers are shown as diamonds. IQR, interquartile range.

On qualitative inspection of colony growth in the live-cell microscopy videos obtained during the nutrient switch, we found that cells roughly doubled in number during the first 2 h in SCD, as is expected from steady-state cells in glucose (Fig. 3 B). After the nutrient switch to SCGE, cells arrested for a period of around 5 h (wild type) but continued to grow in volume. At 5 h after switch, the cells very slowly resumed cell cycle progression. At around 10 h after switch, the cell population appeared to have heterogeneous cell sizes, with large cells that had faced the arrest and small cells born after the arrest. It is important to note here that the limited number of cells that can be analyzed in this assay, in particular at the beginning of the experiment, leads to a large experimental error in the CV of cell volume (Fig. S2). Nevertheless, quantitative analysis confirms the general trends observed in the bulk nutrient switch experiment (Fig. 2 D), namely, that both cell volume and CV of cell volume initially increase after the nutrient switch (Fig. S2). Importantly, we found that the dynamics with which cells resume growth after the nutrient switch are very similar between wild-type and the deletion mutant cells (Fig. S3, A–D). This suggests that differences in the adaptation of cell size are dominated by differences in cell cycle progression, rather than global differences in growth rate due to differences in metabolic adaptation (Fig. S3, A–D). To better understand the postswitch adaptation and its effect on cell volume, it is necessary to consider the cell cycle history of each cell. Accordingly, cells were split into multiple categories (Fig. 3 C), which we then studied separately. All cells that were growing in SCD and faced the nutrient switch to SCGE were categorized as “*switchers*” (Fig. 3 C). At the time of the nutrient switch, the *switchers* could have been either unbudded or budded and were respectively further categorized into “*G1-switchers”* or “*S/G2/M-switchers.*” The specific cell cycle of the *switchers* during which the nutrient switch occurred was called the “*switch-cell-cycle*” (Fig. 3 C, highlighted in yellow). As these *switchers* overcame the postswitch arrest and finished the *switch-cell-cycle,* they divided to give rise to the *first round of daughters*. The *switchers* then entered the *cell-cycle-after-switch-cell-cycle* (Fig. 3 C, highlighted in green), at the end of which they gave rise to the *second round of daughters*. The following rounds of daughters were categorized analogously (Fig. 3 C, highlighted in blue). The rounds of daughters could be further categorized into *daughters of G1-switchers* or *daughters of S/G2/M-switchers.* Notably, the *first round of daughters of S/G2/M-switchers* was the only category apart from the *switchers* themselves that faced the nutrient switch—as buds of the *S/G2/M-switchers*. Albeit complex, this careful categorization of cells was key to identifying specific phenotypes in response to the nutrient switch in the following analysis.

### The nutrient switch causes cell cycle arrests in *switchers* and leads to stronger cell enlargement in *bck2Δ* cells

**Figure 4.**
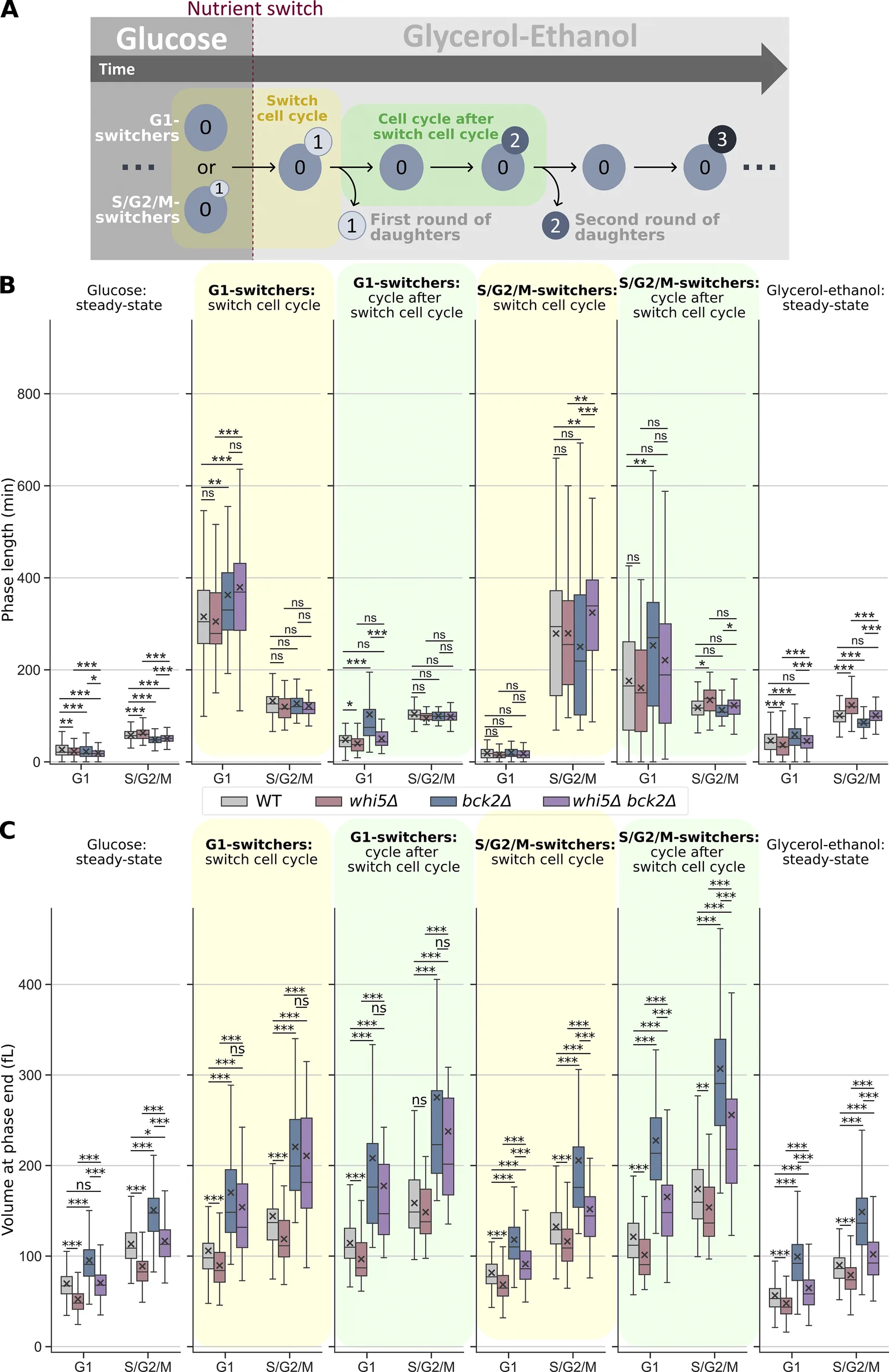
**Nutrient switch causes cell cycle arrests in switchers and leads to stronger cell enlargement in *bck2Δ* cells. (A)** Schematic explanation for cell categories analyzed in this figure. **(B)** Phase lengths are plotted for *switchers’ switch-cell-cycles* (yellow) and *cell cycles after switch-cell-cycle* (green). The outermost left and right panels show steady-state phase lengths for SCD and SCGE, respectively, which each were measured from two independent experiments with constant medium conditions. **(C)** This dataset is also used to determine cell volume at the end of the respective cell cycle phases (fL). Cell volume at the end of S/G2/M is a sum of mother and bud volume at the last frame before division. The number of cells in each box in the steady-state panels is between 450 and 950. For the nutrient switch panels, cells were pooled from three independent experiments. For *G1-switchers’ switch-cell-cycle* G1 (yellow, left): n_WT_ = 144, n_*whi5Δ*_ = 59, n_*bck2Δ*_ = 67, n_*whi5Δbck2Δ*_ = 50. For *G1-switchers’ switch-cell-cycle* S/G2/M (yellow, left): n_WT_ = 132, n_*whi5Δ*_ = 46, n_*bck2Δ*_ = 56, n_*whi5Δbck2Δ*_ = 42. For *S/G2/M-switchers’ switch-cell-cycle* G1 (i.e., the G1 phase before the switch, yellow, right): n_WT_ = 239, n_*whi5Δ*_ = 107, n_*bck2Δ*_ = 116, n_*whi5Δbck2Δ*_ = 115. For *S/G2/M-switchers’ switch-cell-cycle* S/G2/M (yellow, right): n_WT_ = 197, n_*whi5Δ*_ = 66, n_*bck2Δ*_ = 66, n_*whi5Δbck2Δ*_ = 94. For *G1-switchers’ cell-cycle-after-switch-cell-cycle* G1 (green, left): n_WT_ = 120, n_*whi5Δ*_ = 34, n_*bck2Δ*_ = 49, n_*whi5Δbck2Δ*_ = 34. For *G1-switchers’ cell-cycle-after-switch-cell-cycle* S/G2/M (green, left): n_WT_ = 91, n_*whi5Δ*_ = 14, n_*bck2Δ*_ = 39, n_*whi5Δbck2Δ*_ = 26. For *S/G2/M-switchers’ cell-cycle-after-switch-cell-cycle* G1 (green, right): n_WT_ = 164, n_*whi5Δ*_ = 47, n_*bck2Δ*_ = 51, n_*whi5Δbck2Δ*_ = 61. For *S/G2/M-switchers’ cell-cycle-after-switch-cell-cycle* S/G2/M (green, right): n_WT_ = 138, n_*whi5Δ*_ = 45, n_*bck2Δ*_ = 29, n_*whi5Δbck2Δ*_ = 46. Statistical comparisons were performed using two-sided Mann–Whitney U tests. Each strain was compared with WT and *whi5Δbck2Δ* (***: P < 0.001; **: P < 0.01; *: P < 0.05; ns: not significant, P ≥ 0.05). For boxplots in B and C, box limits show the IQR, whiskers extend to the most extreme data points within 1.5× IQR, the median is shown as a line, and the mean is shown as a cross. WT, wild type; IQR, interquartile range.

As described in Fig. 4, we analyzed two specific cell cycles of *switchers*: the *switch-cell-cycle* (Fig. 4 A, highlighted in yellow) and the *cell-cycle-after-switch-cell-cycle* (Fig. 4 A, highlighted in green). *G1-switchers* arrested in G1 during the *switch-cell-cycle* (Fig. 4 B, left, yellow). The following S/G2/M phase of the switch cycle was not strongly affected in length and was similar to that of cells growing exponentially in SCGE. Also, the phase lengths in the next cell cycle of *G1-switchers* (*cell-cycle-after-switch-cell-cycle*, Fig. 4 B, left, green) were similar to those of cells growing exponentially in SCGE. On the other hand, *S/G2/M-switchers* naturally had a normal steady-state G1 length in SCD and arrested in S/G2/M after switch (Fig. 4 B, right, yellow). After completing the *switch-cell-cycle*, they also exhibited a strongly prolonged G1 phase in the next cycle (Fig. 4 B, right, green). However, the duration of the subsequent S/G2/M was close to that of cells exponentially growing in SCGE (Fig. 4 B, green). These results showed that all cells that faced the nutrient switch immediately arrested in the ongoing cell cycle stage. For *S/G2/M-switchers*, the next G1 was also prolonged.

For each strain, the immediate G1 arrest of *G1-switchers* led to an increased cell volume at the end of G1 (Fig. 4 C, left, yellow). The following G1, although short, slightly increased the cell volume further (Fig. 4 C, left, green). Similarly, also for *S/G2/M-switchers* of all strains, the G1 arrest in the *cell-cycle-after-switch-cell-cycle* led to cell enlargement compared with cells growing in constant SCD media (Fig. 4 C, right, green). During these G1 arrests, cells that lacked Bck2 grew more than wild-type cells with similar initial cell volume (Fig. S3, E and F) and therefore exhibited stronger enlargement and a bigger drift from their steady-state sizes as compared to wild-type and *whi5Δ* cells in both categories of *switchers* (Fig. 4 C). These G1 arrests therefore were sufficient to cause significant differences in cell volume at the end of G1 between wild-type and *whi5Δbck2Δ* cells. The G1 arrests also contribute to the diverging CV and mean cell volume observed after switch in the bulk nutrient switch experiment (Fig. 2).

### Cells that face the nutrient switch as buds arrest in their first G1, leading to stronger cell enlargement in *bck2Δ* cells

**Figure 5.**
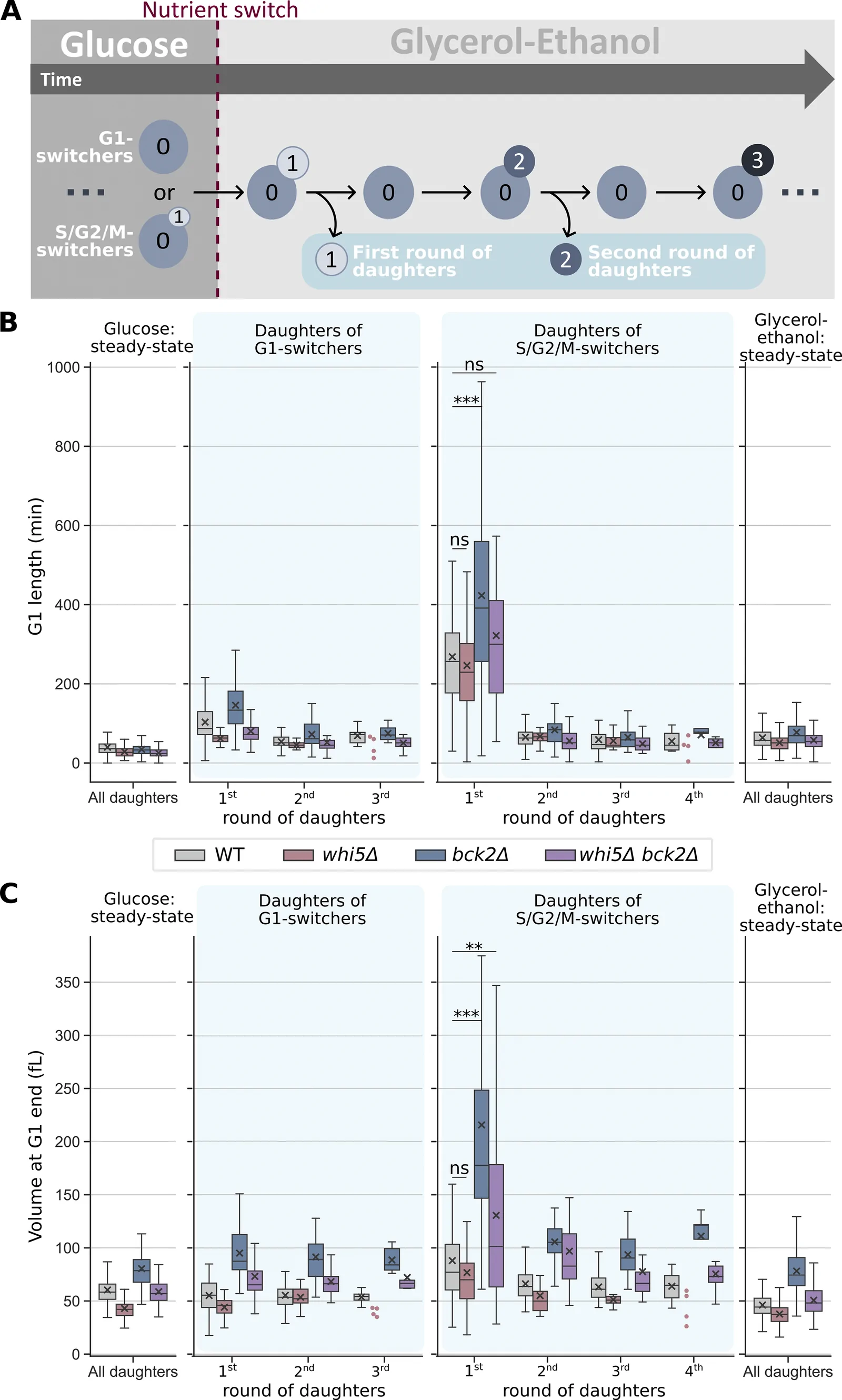
**Cells that face the nutrient switch as buds arrest in their first G1, leading to stronger enlargement in *bck2Δ* cells. (A)** Schematic explanation for cell categories analyzed in this figure. **(B)** G1 lengths are plotted for the *rounds of daughters* (blue) of *G1-switchers* and *S/G2/M-switchers*. The outermost left and right panels show steady-state phase lengths for SCD and SCGE, respectively. For each growth medium, complete cell cycles of daughter cells were pooled from two independent steady-state experiments. **(C)** Same dataset was also used to determine cell volume at the end of the G1 (fL). The number of cells in each box in the steady-state panels is between 198 and 330. For the nutrient switch panels (blue), cells were pooled from three independent experiments. For the *first round of daughters of G1-switchers*: n_WT_ = 103, n_*whi5Δ*_ = 21, n_*bck2Δ*_ = 48, n_*whi5Δbck2Δ*_ = 33. For the *first round of daughters of S/G2/M-switchers*: n_WT_ = 123, n_*whi5Δ*_ = 40, n_*bck2Δ*_ = 34, n_*whi5Δbck2Δ*_ = 50. The next *rounds of daughters* had fewer cells than the first *round*. If a category includes < 5 cells, the individual data points are shown. Two-sided Mann–Whitney U tests were used for statistical analyses (***: P < 0.001; **: P < 0.01; ns: not significant, P ≥ 0.05). For boxplots in B and C, box limits show the IQR, whiskers extend to the most extreme data points within 1.5× IQR, the median is shown as a line, and the mean is shown as a cross. WT, wild type; IQR, interquartile range.

**Figure S4.**
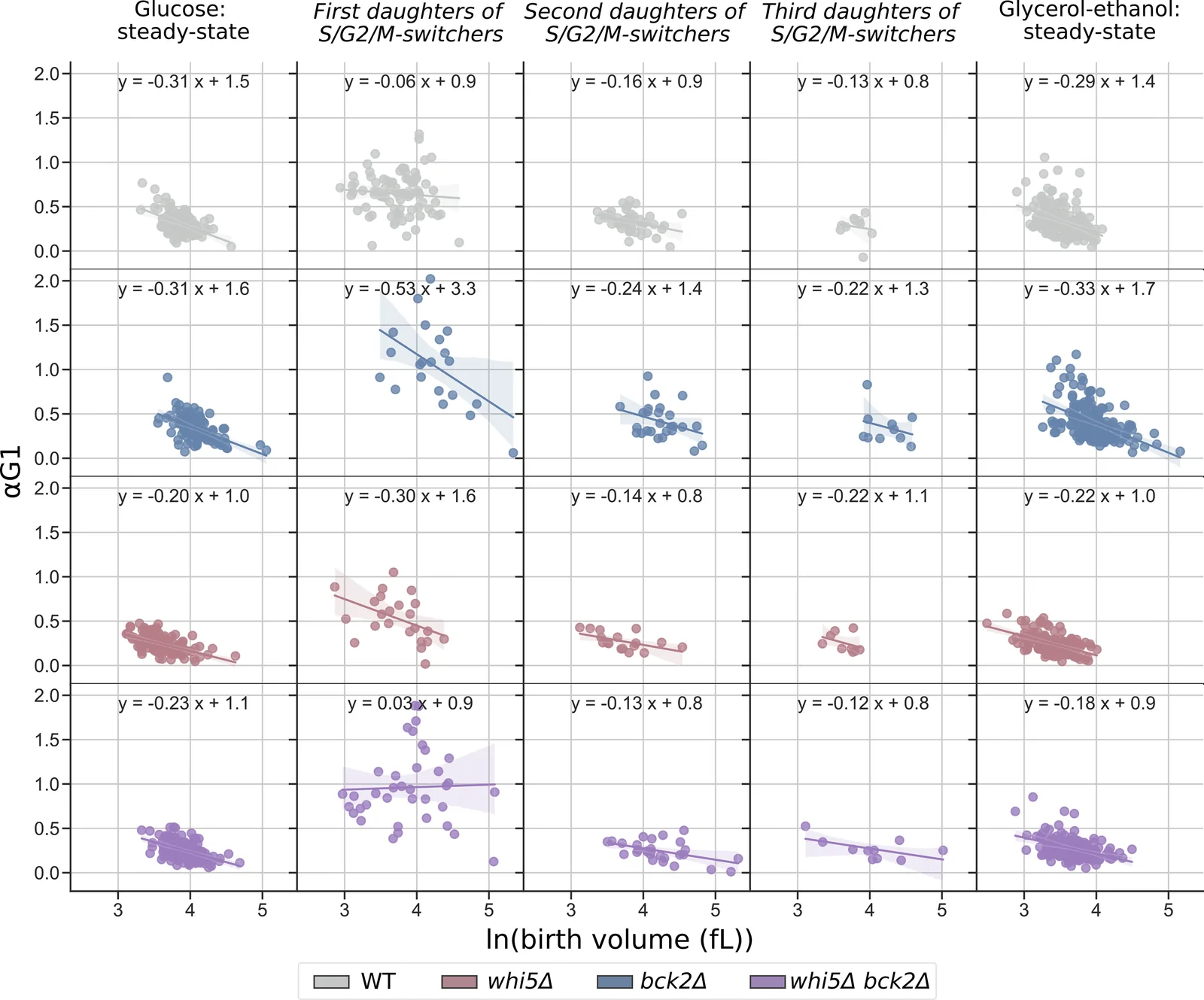
**Cell size homeostasis at the G1/S transition is weaker in the *daughters of S/G2/M-switchers*.** This figure shows the strength of size homeostasis at the G1/S transition in different cell categories assessed as per the method described in Di Talia et al. (2007). The growth rate for exponentially growing cells, α, is the slope of the natural logarithm (in fL) of cell volume plotted against time. For each cell, G1 length is multiplied by α to obtain a growth rate–adjusted G1-duration, αG1. αG1 is plotted against the natural logarithm of birth volume, and a linear regression is performed. A negative slope of the resulting regression line indicates strong cell size homeostasis, whereas a slope closer to 0 indicates weaker cell size homeostasis. Shaded areas show 95% confidence intervals. While steady-state WT daughters (outermost left and outermost right panels) show a steeper negative slope and therefore stronger size homeostasis, the first round of daughters of *S/G2/M-switchers* (WT, second panel) exhibits much weaker size homeostasis. The following rounds of WT daughters show slight recovery in size homeostasis strength. In contrast, the first round of daughters of *whi5Δ* and *bck2Δ S/G2/M-switchers*, but not those of *whi5Δbck2Δ S/G2/M-switchers*, shows stronger size homeostasis. In steady state, *whi5Δ* and *whi5Δbck2Δ* daughters have the lowest G1/S size control efficiency. WT, wild type.

**Figure S5.**
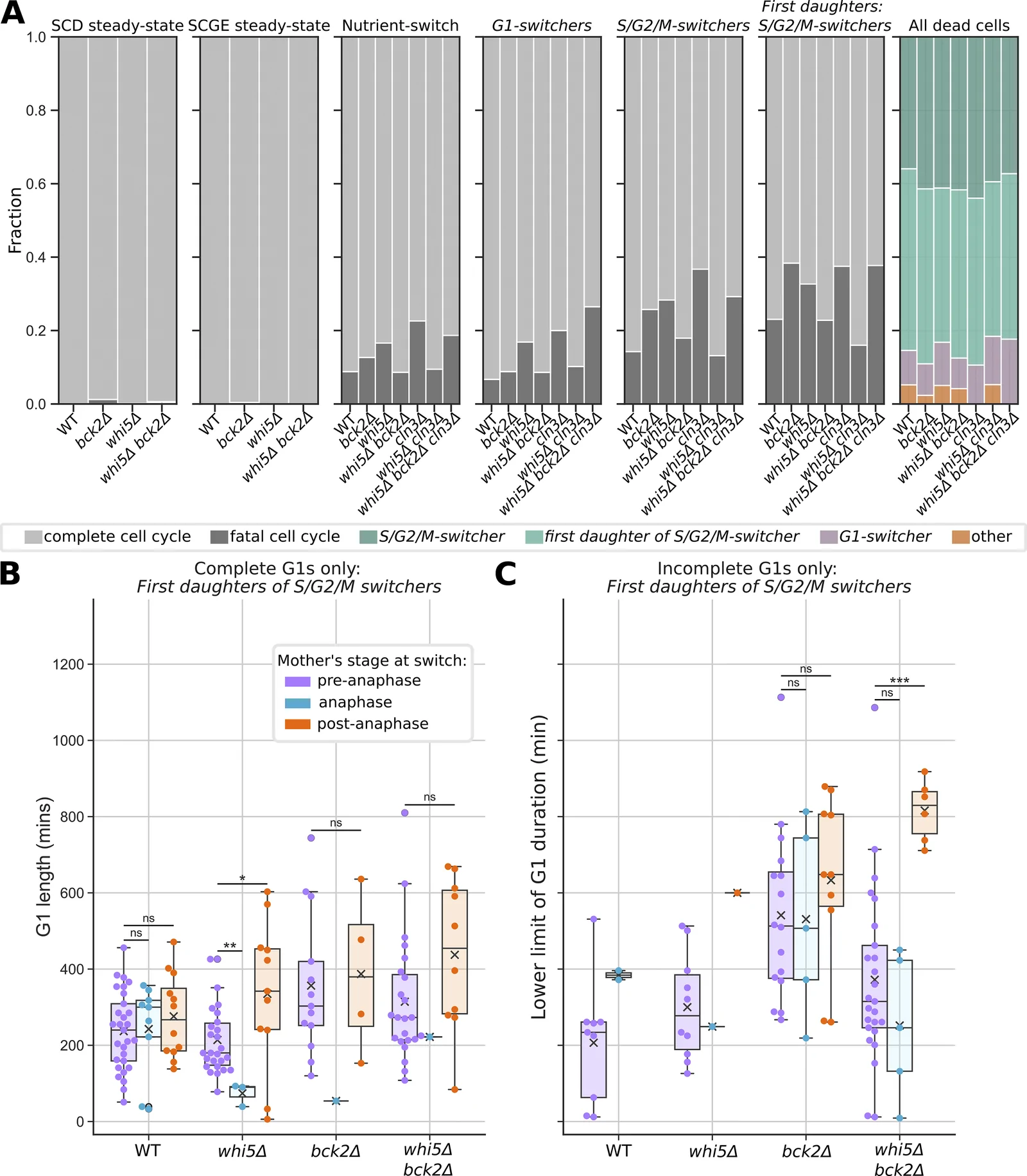
**Analysis of dying cells and arrest phenotypes based on mother cell cycle stage. (A)** For cells pooled from a minimum of two independent nutrient switch experiments, all complete cell cycles and cell cycles during which the cells died were identified. Of this total, the fraction of cell cycles during which cells died (fatal cell cycles, dark gray) and the fraction of cell cycles that were completed (complete cell cycle, light gray) are plotted in a stacked bar plot. The first two panels show the fraction of fatal cycles in steady state in SCD and SCGE. The third panel shows the fraction of fatal cycles during or after the nutrient switch. In the nutrient switch, the single deletions, *whi5Δ*, *cln3Δ*, and *bck2Δ*, and the triple deletion, *whi5Δbck2Δcln3Δ*, had increased fractions of fatal cell cycles as compared to WT. The double deletions, *whi5Δbck2Δ* and *whi5Δcln3Δ*, rescued survival. This may be linked to the double deletions being closer to WT size than the single deletions. Panels four, five, and six show the fraction of fatal cycles if only cell populations of interest are considered: *G1-switchers*, *S/G2/M-switchers*, and *first daughters of S/G2/M-switchers*. In the last panel, all fatal cycles during and after the nutrient switch are pooled. The fraction of dead cells coming from different cell categories is plotted. *S/G2/M-switchers* and their *first daughters* make up the majority of dying cells. **(B and C)** In *bck2Δ* cells, the first daughters of mothers which faced the nutrient switch after anaphase have the strongest arrest phenotypes. We categorized the first round of daughters of *S/G2/M-switchers* by whether the mother was in pre-anaphase (purple), anaphase (blue), or post-anaphase (orange) at the time of the nutrient switch and analyzed the corresponding G1 lengths. The durations of complete and incomplete G1 phases of the first daughters of *S/G2/M-switchers* are plotted in B and C, respectively. Incomplete G1 phases are G1 phases interrupted by the end of the experiment and not by death or exclusion of cells from the analysis. The y axis of C therefore is a lower limit of G1 duration. For *whi5Δbck2Δ* cells, the strongest arrest phenotype, i.e., the longest G1 lengths, was observed for the group whose mothers were in post-anaphase at the time of nutrient switch (B). This was also reflected in the lower limit of G1 durations that we inferred for incomplete G1 phases (C). For *bck2Δ*, we did not observe any complete G1 phases for the group of daughters whose mothers were in post-anaphase at the time of nutrient switch (B) as all of these daughters stayed arrested in G1 until the end of the experiment (C). Significance was tested with two-sided Mann–Whitney U tests comparing each category (***: P < 0.001; **: P < 0.01; *: P < 0.05; ns: not significant, P ≥ 0.05). No statistics are shown in case of a too low sample size. Box limits show the IQR, whiskers extend to the most extreme data points within 1.5× IQR, the median is shown as a line, and the mean is shown as a cross. The individual data points are shown additionally. WT, wild type; IQR, interquartile range.

So far, we have shown that deletion of *BCK2* leads to more enlarged *switchers* during their first G1 after the switch. Apart from the actual *switchers*, also the *first round of daughters* of *S/G2/M-switchers* faced the switch because they were present as buds (Fig. 5 A, highlighted in blue, cell number 1). The *first round of daughters of G1-switchers*, on the other hand, first appeared after the nutrient switch. G1 duration was three- to fourfold longer for the *first round of daughters* of *S/G2/M-switchers*, indicating that they arrested in G1 (Fig. 5 B). This phenomenon was specific for this category of cells, as daughter cells born in the following rounds and those of *G1-switchers* showed G1 durations more similar to steady-state growth on SCGE (Fig. 5 B). Consequently, the *first round of daughters of S/G2/M-switchers* was also larger at G1 end, with *bck2Δ* and *whi5Δbck2Δ* cells again growing more, even at a given starting cell volume (Fig. S3 G). Thus, they show significantly stronger enlargement compared with cells in steady state in SCD media than the other strains (Fig. 5 C). The G1 arrest was also longer for the Bck2 mutant strains as compared to the others (Fig. 5 B). It appears that the cells that faced the switch in S/G2/M, either as mother or bud, carried the memory of the switch into the next cell cycle, leading to a prolonged G1 phase in the next cycle. Moreover, the first daughters of S/G2/M-switchers showed weakened size control (Fig. S4) and lower survival (Fig. S5 A).

### *First round of daughters* of *S/G2/M-switchers* arrests mainly in pre-*Start* G1

**Figure 6.**
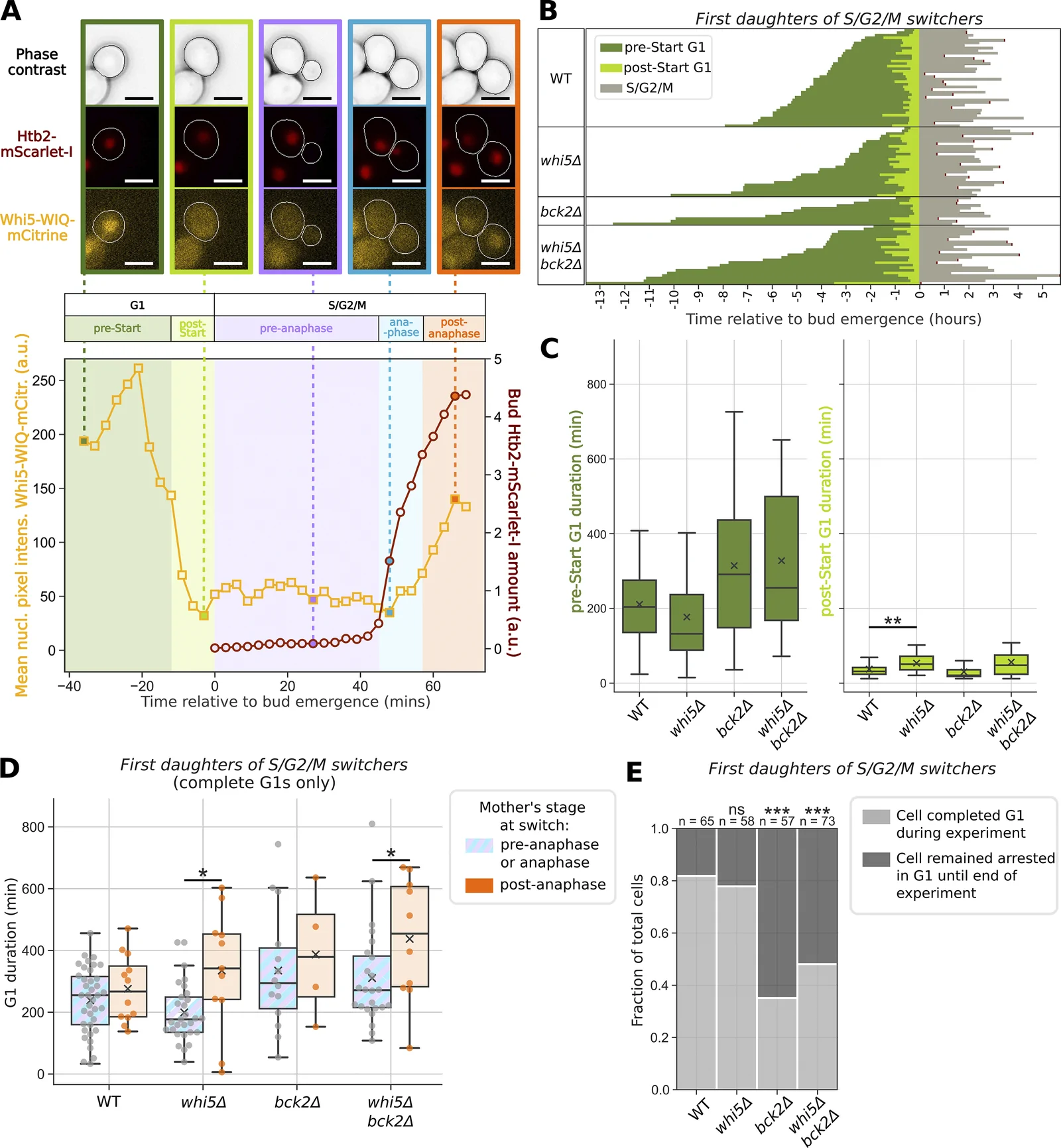
***First round of daughters* of *S/G2/M-switchers* arrests mainly in pre-Start G1.** In *bck2Δ* mutants, the strongest arrest phenotypes are observed in first daughters whose mothers faced the nutrient switch after anaphase. **(A)** Example of cell cycle phase resolution for a steady-state (SCD) WT cell based on two fluorescent signals: mean nuclear pixel intensity of Whi5-WIQ-mCitrine (yellow trace, a.u.) and bud Htb2-mScarlet-I amount (red trace, a.u.), both plotted against time relative to bud emergence. 50% Whi5-WIQ-mCitrine nuclear exit was annotated as *Start*. Entry of Htb2-mScarlet-I into the bud was annotated as start of anaphase. Reentry of Whi5-WIQ-mCitrine into the nucleus was annotated as end of anaphase. Dotted lines connect example images to the time point at which they were acquired. Example images show cells in phase contrast (gray), histone Htb2 tagged with mScarlet-I (red), and Whi5-WIQ tagged with mCitrine (yellow). Cell contours generated with YeaZ in the Cell-ACDC pipeline are shown in white. Scale bars represent 4 µm. **(B)** Cell cycles of *first daughters of S/G2/M-switchers* with complete G1 phases were resolved into pre-Start G1 (dark green), post-Start G1 (light green), and S/G2/M (gray). A categorical heatmap shows cell cycle phases of single cells (as rows), plotted against time relative to bud emergence. Incomplete S/G2/M phases are capped with red ends. Black horizontal lines separate cells of different strains. **(C)** Quantification of pre-Start G1 durations and post-Start durations is plotted as a boxplot. Data were pooled from two independent experiments of live-cell microscopy coupled to nutrient switch. n_WT_ = 38, n_*whi5Δ*_ = 27, n_*bck2Δ*_ = 11, n_*whi5Δbck2Δ*_ = 23. Two-sided Mann–Whitney U tests were used to statistically analyze each strain compared with WT (**: P < 0.01; not significant [P ≥ 0.05], not shown). **(D)** The durations of complete G1 phases of *first daughters of S/G2/M-switchers* are plotted in D. The *first daughters of S/G2/M-switchers* are further categorized on the basis of their mothers’ cell cycle phase at the time of nutrient switch: pre-anaphase or anaphase (purple and blue striped) or post-anaphase (orange). Two-sided Mann–Whitney U tests were used to statistically compare pre-anaphase or anaphase with post-anaphase mother’s stage for each strain (*: P < 0.05; not significant [P ≥ 0.05], not shown). **(E)** Fraction of first daughters of S/G2/M-switchers that complete G1 during the experiment (light gray) and those that stay arrested in G1 until the end of the experiment (cells with incomplete G1 phases, dark gray) is plotted. Incomplete G1 phases are G1 phases interrupted by the end of the experiment and not by death or exclusion of cells from the analysis. Chi-square tests of independence of variables were used for statistical analysis, comparing each strain with WT (***: P < 0.001; ns: not significant, P ≥ 0.05). For boxplots in C and D, box limits show the IQR, whiskers extend to the most extreme data points within 1.5× IQR, the median is shown as a line, and the mean is shown as a cross. The individual data points are additionally shown in D. WT, wild type; IQR, interquartile range.

After showing that *S/G2/M-switchers* and their first daughters exhibit a prolonged first G1 following the nutrient switch, we asked at what point in G1 cells arrest, and whether this arrest depends on the exact cell cycle stage at the time of the nutrient switch. To further characterize this multigenerational arrest phenotype, we employed cell cycle reporters that allowed us to resolve the cell cycle into more specific cell cycle phases. In particular, we used a mCitrine-tagged mutant *WHI5* allele, *WHI5-WIQ*, integrated into the *URA3* locus, while the endogenous *WHI5* gene remained unaltered. The Whi5-WIQ protein is a loss-of-function mutant that does not bind SBF (Travesa et al., 2013) but retains its ability to localize to the nucleus in a cell cycle–dependent manner. In addition, we tagged the histone *HTB2* with the fluorescent protein mScarlet-I. The deletions of interest—Δ*whi5*, Δ*bck2*, and Δ*whi5*Δ*bck2*—were then introduced into this cell cycle reporter strain, and the nutrient switch experiment was repeated. Fluorescently tagged Htb2 allowed us to segment a nuclear mask for each cell. Using the nuclear mask along with cellular segmentation, we could quantify fluorescence signal in three compartments: the whole cell, the nucleus, and the cytoplasm. Whi5 has previously been reported to be nuclear from telophase until *Start*, while CDK activity is low (Taberner et al., 2009). For cell categories of interest, we identified *Start* as the G1 frame at which 50% of Whi5 had exited the nucleus (Doncic et al., 2011) (Fig. 6 A). We used the Htb2-mScarlet-I amount in the bud as a marker for the entry of the dividing nucleus into the bud, which occurs during anaphase in mitosis (Bloom, 2001) (Fig. 6 A). Anaphase was presumed to continue until Whi5-WIQ-mCitrine reentered the nucleus (Taberner et al., 2009) (Fig. 6 A). The nuclear reentry of Whi5-WIQ was annotated as the start of telophase, and subsequent frames were labeled as post-anaphase (Fig. 6 A). Fig. 6 B shows the cell cycle phases of the *first round of daughters* of *S/G2/M-switchers*. After splitting G1 into pre- and post-*Start*, we found that the G1 arrest observed in the *first round of daughters* of *S/G2/M-switchers* was predominantly in pre-S*tart* G1 (dark green areas, Fig. 6 B). Upon quantification (Fig. 6 C), we found that *bck2Δ* and *whi5Δbck2Δ* cells appear to have longer pre-*Start* G1 durations as compared to wild type (albeit not statistically significant).

### In *bck2Δ* cells, the first daughters of mothers that faced the nutrient switch after anaphase have the strongest arrest phenotypes

A previous study by Leitao et al. (Leitao and Kellogg, 2017) has shown that a shift from glucose to glycerol–ethanol media leads to a long arrest for cells that experience the shift in metaphase, but not for anaphase cells. We therefore asked whether the specific cell cycle phase of *S/G2/M-switchers* at the time of the nutrient switch also affects the severity of the G1 arrest in their *first round of daughters*. Thus, we categorized the *first round of daughters* of *S/G2/M-switchers* by whether the mother was in pre-anaphase, anaphase, or post-anaphase at the time of nutrient switch and analyzed the corresponding G1 lengths (Fig. 6 D; and Fig. S5, B and C). Fig. 6 D shows the durations of the G1 phases of the first round of daughters of *S/G2/M-switchers* that completed G1 during the course of the experiment. The fraction of the first round of daughters of *S/G2/M-switchers* that did not complete G1, i.e., remained arrested in G1 until the end of the experiment, is shown in Fig. 6 E, and the lower limits of their G1 durations are plotted in Fig. S5 C. For *bck2Δ* and *whi5Δbck2Δ* cells, the strongest arrest phenotype, i.e., the longest G1 lengths, was observed for the group whose mothers were in post-anaphase at the time of nutrient switch (Fig. 6 D). Especially for *bck2Δ* cells, we could not observe many complete G1 phases for the group of daughters whose mothers were in post-anaphase at the time of nutrient switch (Fig. 6 D) as most of these daughters stayed arrested in G1 until the end of the experiment (Fig. S5, B and C; and Fig. 6 E). In fact, for *bck2Δ* and *whi5Δbck2Δ* cells, the majority of the first round of daughters of *S/G2/M-switchers* did not complete G1 until the end of the experiment (Fig. 6 E). Thus, our data suggest that Bck2 has a nutrient switch–specific function after anaphase, which affects G1 exit in the next generation. Such a role for Bck2 had previously been proposed (Bastajian et al., 2013), where Bck2 was speculated to integrate environmental information into the cell cycle progression decision at multiple cell cycle phase transitions, including M/G1.

Additionally, it has previously been shown that Bck2 promotes cell cycle progression through *Start* by promoting the expression of Cln3, Swi4, and Cln2 (Bastajian et al., 2013). It was also shown that Cln3 is a short-lived protein and depleted after a switch from rich to poor carbon source (Sommer et al., 2021) and that *cln3Δbck2Δ* is inviable due to permanent G1 arrest. One explanation for the extended G1 arrests in Bck2 deletion mutants we identified here could therefore be a postswitch Cln3 depletion, which, coupled with the absence of Bck2, temporarily mimics a *cln3Δbck2Δ* phenotype.

### Bck2, but not Cln3, is critical for size adaptation after a nutrient switch

**Figure 7.**
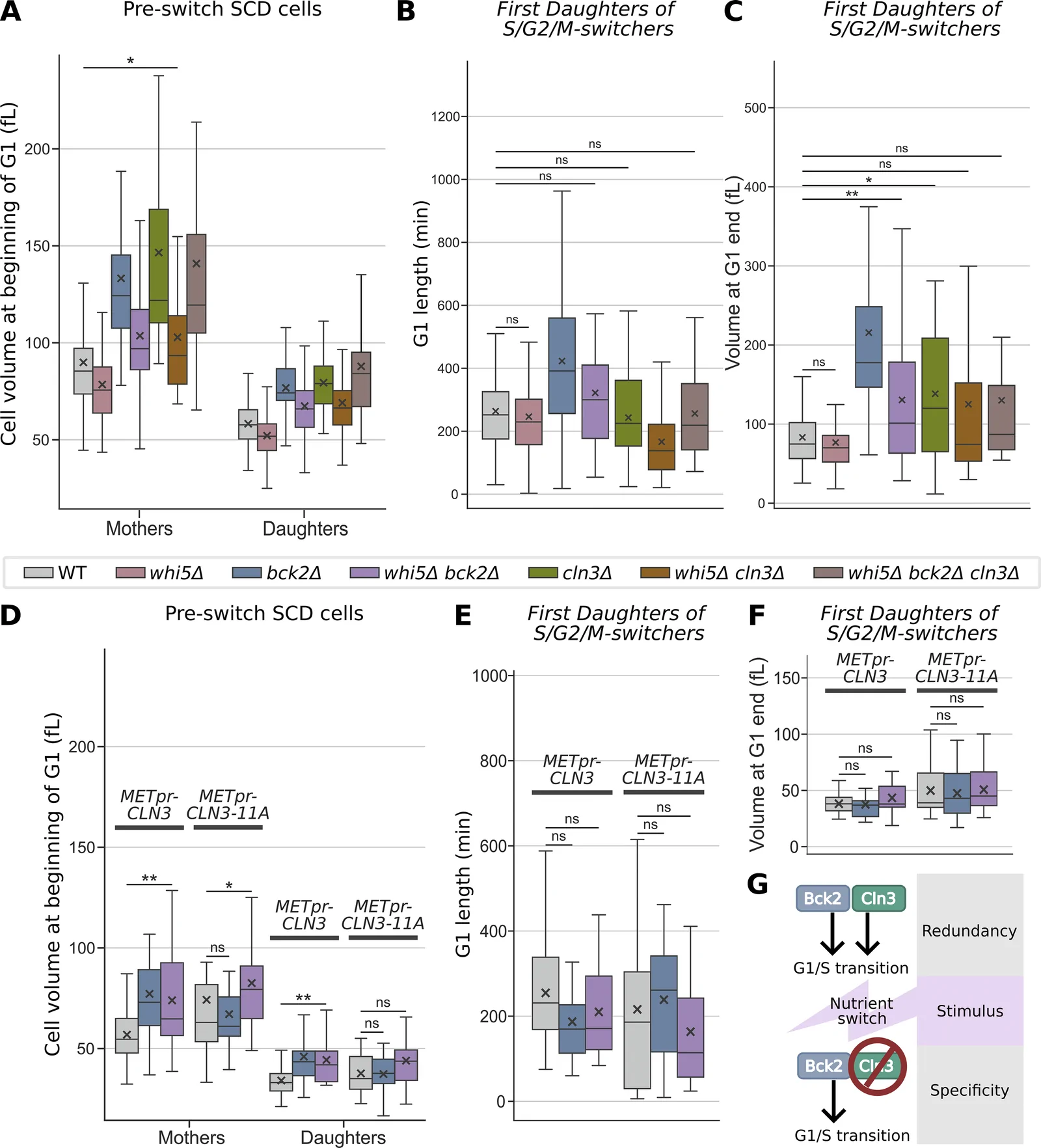
***bck2Δ* cells have a longer G1 arrest and stronger enlargement than *cln3Δ* cells after a nutrient switch. (A)** Cell volume at the beginning of G1 is plotted for daughter (first-generation) and mother (generation > 1st) cells of various strains. For mothers: n_WT_ = 209, n_*whi5Δ*_ = 87, n_*bck2Δ*_ = 95, n_*whi5Δbck2Δ*_ = 95, n_*cln3Δ*_ = 48, n_*whi5Δcln3Δ*_ = 61, n_*whi5Δbck2Δcln3Δ*_ = 34. For daughters: n_WT_ = 164, n_*whi5Δ*_ = 82, n_*bck2Δ*_ = 73, n_*whi5Δbck2Δ*_ = 77, n_*cln3Δ*_ = 44, n_*whi5Δcln3Δ*_ = 55, n_*whi5Δbck2Δcln3Δ*_ = 37. **(B and C)** G1 lengths (B) and cell volumes at G1 end (C) are plotted for the *first daughters* of *S/G2/M-switchers*. The dataset for WT, *whi5Δ*, *bck2Δ*, and *whi5Δbck2Δ* is the same as in Fig. 5. For *cln3Δ*, *whi5Δcln3Δ*, and *whi5Δbck2Δcln3Δ*, cells were pooled from two independent experiments. n_*cln3Δ*_ = 16, n_*whi5Δcln3Δ*_ = 39, n_*whi5Δbck2Δcln3Δ*_ = 11. **(D)** Cell volume at the beginning of G1 is plotted for daughter (first-generation) and mother (generation > 1st) cells of various strains. For *METpr-CLN3* mothers: n_WT_ = 44, n_*bck2Δ*_ = 44, n_*whi5Δbck2Δ*_ = 26. For *METpr-CLN3* daughters: n_WT_ = 36, n_*bck2Δ*_ = 32, n_*whi5Δbck2Δ*_ = 19. For *METpr-CLN3-11A* mothers: n_WT_ = 30, n_*bck2Δ*_ = 22, n_*whi5Δbck2Δ*_ = 30. For *METpr-CLN3-11A* daughters: n_WT_ = 21, n_*bck2Δ*_ = 20, n_*whi5Δbck2Δ*_ = 23. **(E and F)** G1 lengths (E) and cell volumes at G1 end (F) are plotted for the *first daughters* of *S/G2/M-switchers*. Cells were pooled from two independent experiments. For *METpr-CLN3*: n_*WT*_ = 18, n_*bckΔ*_ = 22, n_*whi5Δbck2Δ*_ = 15. For *METpr-CLN3-11A*: n_*WT*_ = 10, n_*bckΔ*_ = 16, n_*whi5Δbck2Δ*_ = 16. **(G)** Schematic illustration of the proposed Bck2 and Cln3 function during steady-state growth and nutrient shift. Two-sided Mann–Whitney U tests were used to statistically analyze each strain compared with WT (P < 0.001 not indicated for clarity; **: P < 0.01; *: P < 0.05; ns: not significant, P ≥ 0.05). For boxplots in A–F, box limits show the IQR, whiskers extend to the most extreme data points within 1.5× IQR, the median is shown as a line, and the mean is shown as a cross. WT, wild type; IQR, interquartile range.

This led us to ask whether similar to *bck2Δ, cln3Δ* also has a strong arrest phenotype after a nutrient switch, or whether, as would be expected if Cln3 is depleted, Cln3 is dispensable for cell size adaptation after the switch. During steady-state growth on SCD, *cln3Δ* cells are larger than wild-type cells, similar to *bck2Δ* (Fig. 7 A). The large size of *bck2Δ* and *cln3Δ* can be partially rescued by an additional deletion of Whi5 (Fig. 7 A). The *whi5Δbck2Δcln3Δ* triple deletion is similar in size to the *bck2Δ* and *cln3Δ* single deletions (Fig. 7 A), which further highlights that the effects of the Whi5, Bck2, and Cln3 deletions are additive.

In contrast, during adaptation to the nutrient switch, *cln3Δ* cells do not have as strong a phenotype as *bck2Δ* cells, in terms of both the G1 length of the first *daughters of S/G2/M-switchers* (Fig. 7 B) and their volume at G1 end (Fig. 7 C). While in steady state, the *whi5Δbck2Δcln3Δ* triple deletion resembles the *bck2Δ* and *cln3Δ* single deletions in size (Fig. 7 A), in the first daughters of S/G2/M-switchers born after the nutrient switch, its size is closer to the *whi5Δbck2Δ* double deletion, not the *bck2Δ* single deletion (Fig. 7 C). This observation is consistent with a nutrient switch–associated depletion of Cln3, rendering Bck2 critical during the nutrient switch.

To further test this hypothesis, we then asked whether the strong *bck2Δ* phenotype during the nutrient switch could be mitigated by induced expression of *CLN3*. Specifically, we expressed either *CLN3* or the stabilized but less active variant *CLN3-11A* from a *MET25* promoter (Schmoller et al., 2015), and investigated how this affects the phenotype of *bck2Δ* and *whi5Δbck2Δ* deletions during the nutrient shift. Consistent with the *MET25* promoter leading to *CLN3* overexpression, all strains showed reduced cell volumes during steady-state growth on SCD (Fig. 7, A and D). Moreover, as predicted by our hypothesis, the constitutive expression of *CLN3* in the *bck2Δ* and *whi5Δbck2Δ* strains largely prevents the enlargement of the *first round of daughters of S/G2/M-switchers* compared with steady-state growth (Fig. 7, E and F).

Taken together, our results are consistent with the model that a depletion of Cln3 during the nutrient shift makes Bck2 temporarily essential (Fig. 7 G), leading to longer G1 arrests and cellular enlargement in *bck2Δ* cells. Importantly, this highlights that unique functions for seemingly redundant proteins can be uncovered by studying single cells in dynamically changing environments.

## Discussion

In nature, yeast cells regularly face changing environments. Cell size control therefore comprises two tasks: size homeostasis, which is the maintenance of narrow size distributions in largely constant environments, and size adaptation, which is the adjustment of cell size to the optimum for a changed environment. So far, most studies on cell size regulation have focused on steady-state conditions. While these studies have been useful for identifying size homeostasis mechanisms and size regulators, they cannot, by design, provide insights into mechanisms of size adaptation. This suggests that studying cell size adaptation in response to nutrient switches may reveal aspects of cell size regulation that were not accessible through steady-state experiments. In particular, we asked whether nutrient switches could give insights into specific functions of cell size regulators that appear redundant in steady-state size control.

The analysis of nutrient switch experiments at the single-cell level is even more complex than that of steady-state experiments. In steady state, key insights can be gained by analyzing individual cell cycles, without tracking cells over multiple generations. In nutrient switch experiments, the history of a cell, specifically the cell cycle stage at the time of the nutrient shift, affects how the cell and its offspring adapt to the new environment (this study and [Irvali et al., 2023]). To understand cell size adaptation, it is therefore crucial to categorize cells based on their cell cycle stage at the time of the nutrient switch before following their progeny over multiple generations. Several studies have investigated nutrient switches with live-cell microscopy in different contexts (Irvali et al., 2023; Bheda et al., 2020; Schuh et al., 2022; Shabestary et al., 2024; Stockwell and Rifkin, 2017). However, none of them performed multigenerational lineage tracking in addition to cell categorization based on the cell cycle stage at the time of the nutrient switch. Here, we built on recent progress in machine-learning approaches and performed the required analysis using the image analysis tools YeaZ and Cell-ACDC (Padovani et al., 2022; Dietler et al., 2020). This enabled us to track complete cell lineages over multiple generations throughout the adaptation to a nutrient switch and create a framework for dealing with the complexity of single-cell categorization.

From bulk experiments, we found that while the simultaneous deletion of two important size regulators, Whi5 and Bck2, hardly affects cell size or cell size homeostasis during steady-state growth in glucose media, it disrupts both after a nutrient switch from glucose to glycerol/ethanol media. Around 10–25 h following a nutrient switch, both wild-type and *whi5Δbck2Δ* cells show a strong increase in the CV of cell volume, indicating weaker size homeostasis. This disruption of size homeostasis is stronger in the *whi5Δbck2Δ* strain. Consistent with our findings, a similar postswitch increase in the CV of cell volume has also been observed in a recent study that analyzes budding yeast adaptation to a nitrogen downshift (Shabestary et al., 2024). Using the cell history–based categorization explained above, we followed single cells through the nutrient switch and found that *bck2Δ* mutants show longer cell cycle arrests after the nutrient switch. Cells that were already budding at the time of the nutrient switch, as well as their first daughters born after the nutrient switch, exhibit the longest cell cycle arrests, the strongest cellular enlargement, and the lowest survival after switch. Interestingly, this implies that cells that experience the nutrient shift in their S/G2/M phase carry some sort of “memory” into their next G1 phase, leading to a delay in cell cycle progression. While the molecular origin of this memory remains to be identified, one possibility is that the nutrient shift and the associated changes in growth rate and cell cycle progression alter the concentration of regulatory proteins up to the next cell cycle. This type of mechanism has been proposed to integrate environmental inputs in yeast (Qu et al., 2019) and mammalian cells (Min et al., 2020) to control following cell cycle phase transitions. In our case, the memory seems to be limited to a single cell cycle, since the following rounds of daughters closely resemble those in exponential growth in terms of cell cycle phase durations and cell size.

In contrast to steady-state conditions, where deletion of the G1/S activators *BCK2* and *CLN3* leads to comparable size phenotypes, *cln3Δ* cells show a much weaker phenotype in response to the nutrient shift. Moreover, an additional deletion of *CLN3* in the *whi5Δbck2Δ* strain does not affect the postswitch arrest or enlargement phenotype. Taken together, our findings suggest that while Cln3 and Bck2 have an apparently redundant role in steady-state size control, Bck2 fulfills a specific function during nutrient adaptation. One intriguing explanation for this is that Cln3 may be depleted after the nutrient switch, as observed by Sommer et al. (2021). Following this lead, we found that expressing *CLN3* from a constitutive *MET25* promoter rescues the cellular enlargement of *bck2Δ* cells during the nutrient shift. We therefore propose that the shared role for Bck2 and Cln3 in activating the G1/S transition serves as a potential “backup plan” for G1 exit when Cln3 is depleted due to changing nutrient environments (Fig. 7 G).

It is also known that Bck2 interacts with multiple proteins involved in nutrient sensing and promotes expression of cell cycle regulators (Bastajian et al., 2013). One of the proteins it interacts with is Tpd3, which is the scaffolding subunit of the protein phosphatase PP2A (Bastajian et al., 2013). PP2A is part of the cascade by which two nutrient-sensing pathways, TOR and PKA, regulate *Start* (Ewald, 2018; García-Blanco et al., 2019; Pedruzzi et al., 2003). This interaction places Bck2 directly at the link between nutrient-dependent growth signaling and cell cycle progression. Thus, a role of Bck2 in nutrient signaling could also contribute to the longer cell cycle arrests and stronger cellular enlargement observed in *bck2Δ* cells after a nutrient switch event. Additionally, Bck2 has been shown to contribute to cell wall maintenance during growth and to the cell wall stress response (Ferrezuelo et al., 2009; Kuravi et al., 2011). It has been shown to induce the expression of nearly 20 proteins involved in cell wall structure and biosynthesis (Ferrezuelo et al., 2009) and is associated with the activation of the MAP kinase Mpk1 in the Pkc1-dependent cell wall integrity pathway (Kuravi et al., 2011). Therefore, it cannot be ruled out that the increased importance of Bck2 after the nutrient switch stems from its role in the maintenance of cell wall integrity.

In conclusion, our work here provides new insights into size adaptation to nutrient challenges. We show that the apparent redundancy between the roles of Bck2 and Cln3 at the G1/S transition is specific to steady-state growth and Bck2 becomes the more crucial G1/S transition activator following a nutrient switch (Fig. 7 G). More generally, our work suggests that studying cell size regulation in conditions other than steady state is key to revealing unique roles for apparently redundant size regulators. We therefore expect that using similar approaches for a broader range of mutants and perturbations in yeast and other organisms will give new mechanistic insights into the processes underlying size homeostasis and adaptation.

## Materials and methods

### Yeast strains

All *S. cerevisiae* strains used in this study are haploid derivatives of W303. They were constructed using standard methods and sequence information obtained from SGD (Engel et al., 2025). They were verified by sequencing. A full genotypic description of the strains is available in Table S1. Yeast strains are available upon reasonable request.

### RNA extraction and RT-qPCR

Cells were grown in 50 ml SCD for at least 17 h and maintained at OD_600nm_ <1 before harvest. At OD_600nm_ >0.1, total RNA was extracted using the RNA extraction protocol of YeaStar RNA Kit (Zymo Research). The RNA was treated with TURBO DNase enzyme (Thermo Fisher Scientific) as per the manufacturer’s protocol. The quality of the RNA was examined in an RNA gel, and the concentration and purity were checked using the NanoDrop OneC spectrophotometer from Thermo Fisher Scientific. 1 µg of RNA was reverse-transcribed with random primers and the high-capacity cDNA reverse transcription kit from Thermo Fisher Scientific. The resulting cDNA was diluted 1:10 fold using double-distilled water. qPCR was performed using 2 μl of these dilutions as template. Target-specific primers and SsoAdvanced Universal SYBR Green Supermix (Bio-Rad) were used for qPCRs in 96-well plates (Roche LightCycler 480 Multiwell Plate 96). For each biological replicate, mean Cq values of target genes were calculated by averaging across at least three technical replicates. The mean Cq value of the reference gene, *ACT1*, was subtracted from the mean Cq value of *WHI5* to calculate normalized *WHI5* Cq values (ΔCq). The *WHI5* ΔCq value of wild type was then subtracted from that of the strains of interest to obtain the *WHI5* ΔΔCq value for each strain. The relative *WHI5* mRNA concentration was calculated using the following formula:

$$\text{relative}\;\text{mRNA}\;\text{concentration}=2^{-\left(\Delta \Delta \text{Cq}\right)}.$$

This process was repeated for five different RNA extractions (experimental replicates). These were plotted as a boxplot in Fig. 1 B. For *WHI5*, the forward primer used had the 5′-3′ sequence 5′-ACA​AGA​CAC​TTC​CCG​AGC​TG-3′ and the reverse primer used had the 5′-3′ sequence 5′-CGC​GTC​TGC​ACT​ATC​TGG​AA-3′. For *ACT1*, the forward primer used had the 5′-3′ sequence 5′-AGT​TGC​CCC​AGA​AGA​ACA​CC-3′ and the reverse primer used had the 5′-3′ sequence 5′-GGA​CAA​AAC​GGC​TTG​GAT​GG-3′ (Claude et al., 2021).

### Single-molecule fluorescence in situ hybridization

smFISH was performed using WHI5-mRNA-specific Stellaris FISH probes from Biosearch Technologies. The targeted probes consisted of thirty-seven 18-nucleotide-long oligonucleotides, each labeled with the Quasar-570 dye. The smFISH protocol for *S. cerevisiae* described on www.biosearchtech.com/stellarisprotocols was used for sample preparation. Cells were grown for a minimum of 17 h in 50 ml SCD and maintained at OD_600nm_ <1. At an OD_600nm_ between 0.3 and 0.5, 45 ml of these cultures was fixed by application of 4% formaldehyde (final concentration) for a period of 45 min. The cells were centrifuged at 1,600 *g* for 4 min and washed twice, each time with 1 ml of ice-cold fixation buffer comprising 1.2  M sorbitol (Sigma-Aldrich) and 0.1 M K_2_HPO_4_ (Sigma-Aldrich) at pH 7.5. Cells were then incubated in 1 ml of fixation buffer containing 6.25 µg Zymolyase (Biomol) for 55 min at 30°C. After two more washes with ice-cold fixation buffer, the fixed and digested cells were stored overnight in 70% ethanol at 4°C. On the next day, 300 μl of cells in 70% ethanol was centrifuged (400 *g*, 5 min) and resuspended in 100 μl of Stellaris RNA FISH hybridization buffer (Biosearch Technologies) containing 10% vol/vol formamide and 125 mM smFISH probes. These cells were incubated overnight in the dark at 30°C to allow for hybridization of smFISH probes to mRNA. After overnight hybridization, cells were washed with Stellaris RNA FISH wash buffer A (Biosearch Technologies) containing 10% vol/vol formamide and resuspended in 1 ml Stellaris RNA FISH wash buffer A (Biosearch Technologies) containing 10% vol/vol formamide and 5 ng/ml DAPI stain. Cells were incubated in the DAPI staining solution for 30 min at 30°C, before being washed with Stellaris RNA FISH wash buffer B (Biosearch Technologies). Washed cells were mounted onto glass slides in Vectashield mounting medium (Vector Laboratories) for the first replicate and in ProLong Gold mounting medium (Thermo Fisher Scientific) for the second replicate due to unavailability of Vectashield. The prepped smFISH samples were imaged on a Zeiss LSM 800 microscope using a 63×/1.4 NA oil immersion objective and an Axiocam 506 camera. The Zen 2.3 software was used to acquire multicolor z-stacks containing 20–25 z-slices at 240-nm intervals. Bright-field images were acquired with the TL LED using an exposure time of 140 ms at 6% of maximum intensity. For imaging Quasar-570, an exposure time of 5 s to illumination by a 530-nm LED at 50% intensity was used. DAPI was imaged with an exposure time of 130 ms and illumination at 385 nm and 30% intensity.

## Data analysis for smFISH

Cells were segmented in bright field using the YeaZ neural network (Dietler et al., 2020) in the Cell-ACDC interface (Padovani et al., 2022). After manual correction of segmentation masks and annotation of bud–mother relationships in Cell-ACDC, the smFISH fluorescence spots were counted using an early version of SpotMAX (Padovani et al., 2026), described in detail in Chatzitheodoridou et al. (2024); Kukhtevich et al. (2022). For each strain and experimental replicate, the following settings of the custom routine were adjusted to optimize spot detection. The 3D Gaussian filter was applied with a Sigma in the range of 0.3–2 voxels. The “threshold_triangle” automatic thresholding algorithm was used for instance segmentation of the spot signal. The “effsize_glass_s” filter was used to filter for valid peaks using an effect size threshold in the range of 1–2.1. Spots were also filtered for size with the lower limit ranging from 1.6 to 2 pixels and the upper limit ranging from 4 to 20 pixels.

Spot detection was followed by categorization of cells into one of three different cell cycle stages: G1, S, and G2/M. Unbudded cells with a single nucleus were categorized into G1 stage. Budded cells with a bud volume-to-mother volume ratio of <0.3 were categorized as S stage. The remaining budded cells were categorized as G2/M cells. Since Whi5 transcription peaks in the S phase, Fig. 1 C shows the average number of *WHI5* mRNA in S-phase cells.

## Bulk nutrient switch experiments

Yeast cells were inoculated into 3 ml Yeast Peptone Medium containing 2% glucose (YPD) and incubated at 30°C in a shaking incubator at 250 rpm (Infors, Ecotron) for at least 4 h. Then, cells were washed and inoculated into 50 ml SCD at a starting OD_600nm_ of 0.0001 and incubated overnight at 30°C under shaking conditions (250 rpm). Cells were grown for a minimum of 17 h in 50 ml SCD and maintained at OD_600nm_ <1. At OD_600nm_ between 0.5 and 0.6, cells were washed with and inoculated in SCGE at a starting OD_600nm_ of 0.01 and incubated under the same conditions (30°C, 250 rpm). Starting from the time of inoculation in SCGE, OD_600nm_ and Coulter counter measurements were performed at 1- to 1.5-h intervals until 28.5 h had passed since the nutrient switch. The same measurements were also performed after 48 and 72 h had passed since the nutrient switch. For the measurements taken before the 30-h time point, culture OD_600nm_ was always below 1. For measurements taken 48 and 72 h after the nutrient switch, once the cultures grew to an OD_600nm_ of 0.1, appropriate dilutions were made to maintain the OD_600nm_ between 0.1 and 1. The complete time course of measurements was achieved in sections in three different experiments of two replicates each. Therefore, overlapping time points in the plot have four data points. The CV was calculated from the Coulter counter size distribution with the formula:

$$\begin{array}{c} \text{CV}\;\text{of}\;\text{cell}\;\text{volume}=\text{Standard}\;\text{deviation}\;\text{in}\;\text{cell}\;\text{volume}/\\ \text{Mean}\;\text{cell}\;\text{volume} \end{array}$$

For statistically comparing the two strains, we divided the time course into three 10-h intervals and grouped the remaining time points together (Fig. 2, B and D). We used the Shapiro–Wilk normality tests to confirm that the measurements are normally distributed within each time interval. For each time interval, we performed a mixed ANOVA test, with time as the within-subjects factor and strain as the between-subjects factor for classification. The interaction between the two factors was not significant, and therefore, we could interpret the effects of each factor individually. The P values obtained for each time interval based on the between-subjects factor, i.e., strain, were represented in statistical annotations in the figure (Fig. 2, B and D).

### Live-cell microscopy

#### Culture conditions

Yeast cells were inoculated into 3 ml YPD and incubated at 30°C in a shaking incubator at 250 rpm (Infors, Ecotron) for at least 5 h. After 5 h, cells were washed and inoculated into 50 ml SCD or 2% glycerol and 1% ethanol (SCGE). The cells were allowed to grow for at least 16 h and maintained at OD_600nm_ <1. They were then diluted to OD_600nm_ = 0.1 for microscopy. An analogous strategy was used for the experiments with constitutive Met-inducible expression of Cln3 (Fig. 7, D–F), only that cells were instead immediately inoculated into SCD-Met media.

#### Microscopy setup

Live-cell time-lapse microscopy was performed using a Nikon Eclipse Ti-E microscope in combination with a custom microfluidics device, described in detail in Kukhtevich et al. (2022). The microfluidics device consisted of a polydimethylsiloxane replica with functional structures bonded to a glass coverslip. This created a cavity that could trap cells and limit colony growth to the *XY*-plane. Growth medium was pumped through the microfluidics device at a constant rate of 20 μl/min to sustain the growth of cells in the device during the imaging process. For steady-state experiments, filtered SCD or SCGE was used for the entire duration of the experiment. For nutrient switch experiments, cells were grown in SCD (or SCD-Met for experiments with inducible Cln3, Fig. 7, D–F) for the first 2 h of the experiment, and then, the medium was switched to SCGE (SCGE-Met) for the rest of the experiment. The time taken for the medium to be completely switched within the device was calculated to be 30 min, and medium switch was therefore annotated at 2.5 h in the downstream analysis of the live-cell microscopy data. To maximize the rounds of daughters observed per video before crowding of the field of view prevented further analysis, the microscopy experiments were started with only one or 2 cells per position. This limited the number of cells that could be analyzed at the time of the nutrient switch, which was further reduced by repeated categorization.

The epifluorescence microscopy setup included the Nikon Eclipse Ti-E microscope with NIS-Elements software, SPECTRA X light engine illumination, and the Andor iXon Ultra 888 camera. Phase-contrast and fluorescence images were acquired at 3-min intervals via a Plan-Apo λ 100×/1.45 Na Ph3 oil immersion objective. An additional magnification of 1.5× was applied at the time of imaging. The phase-contrast images were acquired using an exposure time of 100 ms. For mCitrine fluorescence imaging, an exposure time of 300 ms was used and the SPECTRA X light engine was used for illumination at 508 nm and 40% power (24.8 mW). mScarlet-I fluorescence was imaged with an exposure time of 200 ms and illumination at 555 nm and 10% power (26 mW). The image acquisition settings were the same across all live-cell microscopy experiments. The incubation temperature of the cells during live-cell microscopy was maintained at 30°C using the objective heater and a custom heatable insertion.

### Data analysis for live-cell microscopy

Live-cell microscopy data were analyzed with the open-source Cell-ACDC software (Padovani et al., 2022; Padovani et al., 2025). In the data preparation step, the images were aligned and cropped to the region of interest. Automated segmentation and tracking were performed on the phase-contrast channel using the YeaZ neural network (Dietler et al., 2020) in the Cell-ACDC interface. The resulting segmentation masks were manually corrected, and pedigrees were assigned in the Cell-ACDC GUI. The annotated and manually corrected dataset could then be exported for downstream analysis.

#### Calculation of mean cell volume and CV of cell volume

For the mean cell volume and CV of cell volume plots (Fig. 1, F and G, respectively), live cells were pooled from the last frames of all imaging positions per strain, to maximize the number of cells in the analysis. The mean cell volume and its CV were calculated from this pooled population. In Fig. 1 G, the bar plot heights are the CV values obtained from the pooled data and the •, ■, and **x** symbols show CV values obtained if the pooled data are split into the underlying experimental replicates. To statistically compare the CV values between strains, bootstrapping was used to randomly sample positions from each strain in 10,000 iterations with replacement. For each combination of positions, live cells in the last frame were pooled and the CV of cell volume was calculated. To compare the bootstrapped CV distributions between strains, the vector of the CV distribution of one strain was subtracted from the vector of the CV distribution of the other. To estimate P values, the 95, 99, and 99.9% confidence intervals were calculated for the thereby obtained difference vector. If the confidence interval of the difference vector contained 0, it was concluded that the CV distributions overlapped considerably for the two strains at the given confidence level and their difference was not significant.

#### Calculation of cell cycle properties

Based on phase-contrast images, cell cycle annotations in Cell-ACDC categorized unbudded cells into the “G1” phase and all budded cells into an umbrella category: the “S/G2/M” phase. The following functions from the Cell-ACDC downstream analysis Jupyter Notebook were used for calculating cell cycle properties such as phase lengths, and volume changes per phase and for retrieving relatives’ data for each cell: cca_functions.calculate_downstream_data, cca_functions.calculate_per_phase_quantities and cca_functions.calculate_relatives_data. From the resulting dataset, multigenerational mother–bud relationships, or pedigrees, could be acquired for cell populations of interest. Each cell cycle and cell cycle phase was labeled as complete or incomplete, based on whether the cell died during that cycle or phase and whether the cycle or phase was interrupted by the start or the end of the experiment. Unless specified otherwise, only complete cycles or complete phases were used for further analysis.

#### Assignment of cell cycle phases using cell cycle reporters

For the cell cycle reporter strain, fluorescently labeled Htb2 allowed us to segment a nuclear mask for each cell. Using this along with cellular segmentation, we could quantify fluorescence signal in three compartments: the whole cell, the nucleus, and the cytoplasm. Comparing Whi5-WIQ-mCitrine signal and Htb2-mScarlet-I signal between these compartments allowed us to further resolve the cell cycle into more specific phases than just G1 and S/G2/M, as described in the Results section. For budded cells, mean pixel intensity was calculated by calculating the sum of the total pixel intensities of mother and bud, and the combined cell area in pixels for mother and bud, and then dividing the total sum pixel intensity by the total area in pixels. The cytoplasm-adjusted nuclear mean pixel intensity of Whi5-WIQ-mCitrine (Fig. 6 A) was calculated by subtracting cytoplasmic mean pixel intensity of Whi5-WIQ-mCitrine from nuclear mean pixel intensity of Whi5-WIQ-mCitrine. Htb2-mScarlet-I amount was background-adjusted using a background ROI created during the data preparation step of the Cell-ACDC analysis pipeline (Fig. 6 A).

For assigning the time point at which Start occurs, nuclear mean pixel intensity of Whi5-WIQ-mCitrine was plotted against time for each cell of interest. The frame at which nuclear mean pixel intensity of Whi5-WIQ-mCitrine started to decrease during G1 was manually identified (*Whi5_export_start_frame*). Similarly, the frame at which nuclear mean pixel intensity of Whi5-WIQ-mCitrine stopped decreasing was manually identified (*Whi5_export_end_frame*). The nuclear mean pixel intensities of Whi5-WIQ-mCitrine at these two frames were retrieved (*Whi5_intensity_export_start, Whi5_intensity_export_end*), and the difference between the two intensities was calculated. The difference was divided by 2 and added to *Whi5_intensity_export_end* to get nuclear mean pixel intensity of Whi5-WIQ-mCitrine when half of Whi5 has been exported from the nucleus (*Whi5_intensity_50%_export)*. Starting from *Whi5_export_start_frame* and proceeding frame by frame, the nuclear mean pixel intensity of Whi5-WIQ-mCitrine was compared with *Whi5_intensity_50%_export.* The first frame where frame-specific nuclear mean pixel intensity of Whi5-WIQ-mCitrine was less than *Whi5_intensity_50%_export* was assigned as the first post-Start frame. All following G1 frames were categorized as ***post-Start G1***, and all previous G1 frames were categorized as ***pre-Start G1***.

Nuclear mean pixel intensity of Whi5-WIQ-mCitrine was also used for assigning the time point at which cells enter telophase. The first frame of nuclear reimport of Whi5-WIQ-mCitrine in S/G2/M cells was manually identified for individual cells in cell categories of interest. All frames including and after this frame were categorized as ***post-anaphase***.

Background-adjusted Htb2-mScarlet-I amount in the bud was used to assign the beginning of anaphase. The first frame showing increasing Htb2-mScarlet-I amount in the bud was manually identified for cells of interest. All frames after and including this frame and before the first telophase frame were categorized as ***anaphase***.

Finally, all S/G2/M frames starting from and including bud emergence and going until the first anaphase frame were assigned to the ***bud emergence to anaphase*** category, spanning S, G2, prophase, and metaphase.

### Statistical analysis

Statistical analysis, except for bootstrapping, was performed using the SciPy package (Virtanen et al., 2020) written in Python. The code is available online in our GitHub repository.

Normality tests were performed using the Shapiro–Wilk test. In most cases, the tests showed that the microscopy data are not normally distributed. For consistency, except for data shown in Fig. 2, we therefore do not assume normality throughout the paper and chose statistical tests accordingly.

### Online supplemental material

Fig. S1 shows extended analyses of the steady-state experiments. Fig. S2 shows an analysis of population-level behavior of cell volume and its variability during the nutrient shift based on single-cell measurements. Fig. S3 shows that the enlargement phenotype of bck2Δ cells is not explained by different durations of metabolic reprogramming or different starting cell volumes. Fig. S4 shows an analysis of cell size homeostasis at the G1/S transition during the nutrient shift. Fig. S5 shows an analysis of cells dying during the nutrient shift and of how the arrest phenotypes depend on mother cell cycle stage. Table S1 shows a list of the budding yeast strains used in this study.

## Supplementary Material

Table S1 shows the strains used in this study.

## Data Availability

Data are available in a permanent BioImage Archive (Hartley et al., 2022) repository: https://doi.org/10.6019/S-BIAD4064. The code used for the analysis is available on the GitHub page https://github.com/SchmollerLab/Nutrient-switch-analysis.

## References

[bib1] Barber, F., A. Amir, and A.W. Murray. 2020. Cell-size regulation in budding yeast does not depend on linear accumulation of Whi5. Proc. Natl. Acad. Sci. USA. 117:14243–14250. 10.1073/pnas.2001255117 PMC7321981 32518113

[bib2] Bastajian, N., H. Friesen, and B.J. Andrews. 2013. Bck2 acts through the MADS box protein Mcm1 to activate cell-cycle-regulated genes in budding yeast. PLoS Genet. 9:e1003507. 10.1371/journal.pgen.1003507 PMC3649975 23675312

[bib3] Bheda, P., D. Aguilar-Gómez, N.B. Becker, J. Becker, E. Stavrou, I. Kukhtevich, T. Höfer, S. Maerkl, G. Charvin, C. Marr, . 2020. Single-cell tracing dissects regulation of maintenance and inheritance of transcriptional reinduction memory. Mol. Cell. 78:915–925.e7. 10.1016/j.molcel.2020.04.016 32392469

[bib4] Bloom, K. 2001. Nuclear migration: Cortical anchors for cytoplasmic dynein. Curr. Biol. 11:R326–R329. 10.1016/S0960-9822(01)00176-2 11369225

[bib5] Cadart, C., S. Monnier, J. Grilli, P.J. Sáez, N. Srivastava, R. Attia, E. Terriac, B. Baum, M. Cosentino-Lagomarsino, and M. Piel. 2018. Size control in mammalian cells involves modulation of both growth rate and cell cycle duration. Nat. Commun. 9:3275. 10.1038/s41467-018-05393-0 PMC6095894 30115907

[bib6] Chadha, Y., A. Khurana, and K.M. Schmoller. 2024. Eukaryotic cell size regulation and its implications for cellular function and dysfunction. Physiol. Rev. 104:1679–1717. 10.1152/physrev.00046.2023 PMC11495193 38900644

[bib7] Chatzitheodoridou, D., D. Bureik, F. Padovani, K.V. Nadimpalli, and K.M. Schmoller. 2024. Decoupled transcript and protein concentrations ensure histone homeostasis in different nutrients. EMBO J. 43:1–28. 10.1038/s44318-024-00227-w PMC11535423 39271795

[bib8] Chen, Y., G. Zhao, J. Zahumensky, S. Honey, and B. Futcher. 2020. Differential scaling of gene expression with cell size May explain size control in budding yeast. Mol. Cell. 78:359–370.e6. 10.1016/j.molcel.2020.03.012 PMC7963363 32246903

[bib9] Claude, K.-L., D. Bureik, D. Chatzitheodoridou, P. Adarska, A. Singh, and K.M. Schmoller. 2021. Transcription coordinates histone amounts and genome content. Nat. Commun. 12:4202. 10.1038/s41467-021-24451-8 PMC8270936 34244507

[bib10] Costanzo, M., J.L. Nishikawa, X. Tang, J.S. Millman, O. Schub, K. Breitkreuz, D. Dewar, I. Rupes, B. Andrews, and M. Tyers. 2004. CDK activity antagonizes Whi5, an inhibitor of G1/S transcription in yeast. Cell. 117:899–913. 10.1016/j.cell.2004.05.024 15210111

[bib11] D’Ario, M., R. Tavares, K. Schiessl, B. Desvoyes, C. Gutierrez, M. Howard, and R. Sablowski. 2021. Cell size controlled in plants using DNA content as an internal scale. Science. 372:1176–1181. 10.1126/science.abb4348 34112688

[bib12] de Bruin, R.A.M., W.H. McDonald, T.I. Kalashnikova, J. Yates, and C. Wittenberg. 2004. Cln3 activates G1-Specific transcription via phosphorylation of the SBF bound repressor Whi5. Cell. 117:887–898. 10.1016/j.cell.2004.05.025 15210110

[bib13] Di Como, C.J., H. Chang, and K.T. Arndt. 1995. Activation of *CLN1* and *CLN2* G _1_ cyclin gene expression by BCK2. Mol. Cell. Biol. 15:1835–1846. 10.1128/MCB.15.4.1835 PMC230409 7891677

[bib14] Di Talia, S., J.M. Skotheim, J.M. Bean, E.D. Siggia, and F.R. Cross. 2007. The effects of molecular noise and size control on variability in the budding yeast cell cycle. Nature. 448:947–951. 10.1038/nature06072 17713537

[bib15] Dietler, N., M. Minder, V. Gligorovski, A.M. Economou, D.A.H.L. Joly, A. Sadeghi, C.H.M. Chan, M. Koziński, M. Weigert, A.-F. Bitbol, and S.J. Rahi. 2020. A convolutional neural network segments yeast microscopy images with high accuracy. Nat. Commun. 11:5723. 10.1038/s41467-020-19557-4 PMC7665014 33184262

[bib16] Doncic, A., M. Falleur-Fettig, and J.M. Skotheim. 2011. Distinct interactions select and maintain a specific cell fate. Mol. Cell. 43:528–539. 10.1016/j.molcel.2011.06.025 PMC3160603 21855793

[bib17] Engel, S.R., S. Aleksander, R.S. Nash, E.D. Wong, S. Weng, S.R. Miyasato, G. Sherlock, and J.M. Cherry. 2025. Saccharomyces genome database: Advances in genome annotation, expanded biochemical pathways, and other key enhancements. Genetics. 229:iyae185. 10.1093/genetics/iyae185 PMC11912841 39530598

[bib18] Epstein, C.B., and F.R. Cross. 1994. Genes that can bypass the CLN requirement for Saccharomyces cerevisiae cell cycle START. Mol. Cell. Biol. 14:2041–2047. 10.1128/mcb.14.3.2041-2047.1994 PMC358564 8114735

[bib19] Ewald, J.C. 2018. How yeast coordinates metabolism, growth and division. Curr. Opin. Microbiol. 45:1–7. 10.1016/j.mib.2017.12.012 29334655

[bib20] Ferrezuelo, F., M. Aldea, and B. Futcher. 2009. Bck2 is a phase-independent activator of cell cycle-regulated genes in yeast. Cell Cycle. 8:239–252. 10.4161/cc.8.2.7543 19158491

[bib21] García-Blanco, N., A. Vázquez-Bolado, and S. Moreno. 2019. Greatwall-endosulfine: A molecular switch that regulates PP2A/B55 protein phosphatase activity in dividing and quiescent cells. Int. J. Mol. Sci. 20:6228. 10.3390/ijms20246228 PMC6941129 31835586

[bib22] Garmendia-Torres, C., O. Tassy, A. Matifas, N. Molina, and G. Charvin. 2018. Multiple inputs ensure yeast cell size homeostasis during cell cycle progression. Elife. 7:e34025. 10.7554/eLife.34025 PMC6085122 29972352

[bib23] Ginzberg, M.B., N. Chang, H. D’Souza, N. Patel, R. Kafri, and M.W. Kirschner. 2018. Cell size sensing in animal cells coordinates anabolic growth rates and cell cycle progression to maintain cell size uniformity. Elife. 7:e26957. 10.7554/eLife.26957 PMC6031432 29889021

[bib24] Hall, D.D., D.D. Markwardt, F. Parviz, and W. Heideman. 1998. Regulation of the Cln3-Cdc28 kinase by cAMP in Saccharomyces cerevisiae. EMBO J. 17:4370–4378. 10.1093/emboj/17.15.4370 PMC1170770 9687505

[bib63] Hartley, M., G.J. Kleywegt, A. Patwardhan, U. Sarkans, J.R. Swedlow, and A. Brazma. 2022. The BioImage Archive – Building a Home for Life-Sciences Microscopy Data. Journal of Molecular Biology. 434. 167505. 10.1016/j.jmb.2022.167505 35189131

[bib25] Irvali, D., F.P. Schlottmann, P. Muralidhara, I. Nadelson, K. Kleemann, N.E. Wood, A. Doncic, and J.C. Ewald. 2023. When yeast cells change their mind: Cell cycle “Start” is reversible under starvation. EMBO J. 42:e110321. 10.15252/embj.2021110321 PMC9841329 36420556

[bib26] Johnston, G.C., J. Pringle, and L. Hartwell. 1977. Coordination of growth with cell division in the yeast *Saccharomyces cerevisiae*. Exp. Cell Res. 105:79–98. 10.1016/0014-4827(77)90154-9 320023

[bib27] Kõivomägi, M., M.P. Swaffer, J.J. Turner, G. Marinov, and J.M. Skotheim. 2021. G_1_ cyclin-Cdk promotes cell cycle entry through localized phosphorylation of RNA polymerase II. Science. 374:347–351. 10.1126/science.aba5186 PMC8608368 34648313

[bib28] Kukhtevich, I.V., M. Rivero-Romano, N. Rakesh, P. Bheda, Y. Chadha, P. Rosales-Becerra, S. Hamperl, D. Bureik, S. Dornauer, C. Dargemont, . 2022. Quantitative RNA imaging in single live cells reveals age-dependent asymmetric inheritance. Cell Rep. 41:111656. 10.1016/j.celrep.2022.111656 36384120

[bib29] Kuravi, V.K., C. Kurischko, M. Puri, and F.C. Luca. 2011. Cbk1 kinase and Bck2 control MAP kinase activation and inactivation during heat shock. Mol. Biol. Cell. 22:4892–4907. 10.1091/mbc.e11-04-0371 PMC3237631 22031291

[bib30] Leitao, R.M., and D.R. Kellogg. 2017. The duration of mitosis and daughter cell size are modulated by nutrients in budding yeast. J. Cell Biol. 216:3463–3470. 10.1083/jcb.201609114 PMC5674877 28939614

[bib31] Lengefeld, J., C.-W. Cheng, P. Maretich, M. Blair, H. Hagen, M.R. McReynolds, E. Sullivan, K. Majors, C. Roberts, J.H. Kang, . 2021. Cell size is a determinant of stem cell potential during aging. Sci. Adv. 7:eabk0271. 10.1126/sciadv.abk0271 PMC8589318 34767451

[bib32] Manohar, S., and G.E. Neurohr. 2024. Too big not to fail: Emerging evidence for size-induced senescence. FEBS J. 291:2291–2305. 10.1111/febs.16983 37986656

[bib33] Manukyan, A., J. Zhang, U. Thippeswamy, J. Yang, N. Zavala, M.P. Mudannayake, M. Asmussen, C. Schneider, and B.L. Schneider. 2008. Ccr4 alters cell size in yeast by modulating the timing of CLN1 and CLN2 expression. Genetics. 179:345–357. 10.1534/genetics.108.086744 PMC2390614 18493058

[bib34] Miller, K.E., C. Vargas-Garcia, A. Singh, and J.B. Moseley. 2023. The fission yeast cell size control system integrates pathways measuring cell surface area, volume, and time. Curr. Biol. 33:3312–3324.e7. 10.1016/j.cub.2023.06.054 PMC10529673 37463585

[bib35] Min, M., Y. Rong, C. Tian, and S.L. Spencer. 2020. Temporal integration of mitogen history in mother cells controls proliferation of daughter cells. Science. 368:1261–1265. 10.1126/science.aay8241 PMC8363187 32241885

[bib36] Neurohr, G.E., R.L. Terry, J. Lengefeld, M. Bonney, G.P. Brittingham, F. Moretto, T.P. Miettinen, L.P. Vaites, L.M. Soares, J.A. Paulo, . 2019. Excessive cell growth causes cytoplasm dilution and contributes to senescence. Cell. 176:1083–1097.e18. 10.1016/j.cell.2019.01.018 PMC6386581 30739799

[bib38] Padovani, F., B. Mairhörmann, P. Falter-Braun, J. Lengefeld, and K.M. Schmoller. 2022. Segmentation, tracking and cell cycle analysis of live-cell imaging data with Cell-ACDC. BMC Biol. 20:174. 10.1186/s12915-022-01372-6 PMC9356409 35932043

[bib39] Padovani, F., T. Stegmaier, B. Mairhörmann, and K.M. Schmoller. 2025. Analysis of multidimensional microscopy data using Cell-ACDC. J. Vis. Exp. 2025:e68954. 10.3791/68954 41284619

[bib64] Padovani, F., I. Čavka, A.R.R. Neves, C.P. López, N. Al-Refaie, L. Bolcato, D. Chatzitheodoridou, Y. Chadha, X.A. Su, J. Lengefeld, . 2026. SpotMAX: A generalist framework for multi-dimensional automatic spot detection and quantification. Sci. Adv. 12:eadw3811. 10.1126/sciadv.adw3811 PMC13524050 42664352

[bib40] Parviz, F., and W. Heideman. 1998. Growth-independent regulation of CLN3 mRNA levels by nutrients in Saccharomyces cerevisiae. J. Bacteriol. 180:225–230. 10.1128/JB.180.2.225-230.1998 PMC106875 9440509

[bib41] Pedruzzi, I., F. Dubouloz, E. Cameroni, V. Wanke, J. Roosen, J. Winderickx, and C. De Virgilio. 2003. TOR and PKA signaling pathways converge on the protein kinase Rim15 to control entry into G0. Mol. Cell. 12:1607–1613. 10.1016/S1097-2765(03)00485-4 14690612

[bib43] Qu, Y., J. Jiang, X. Liu, P. Wei, X. Yang, and C. Tang. 2019. Cell cycle inhibitor Whi5 records environmental information to coordinate growth and division in yeast. Cell Rep. 29:987–994.e5. 10.1016/j.celrep.2019.09.030 31644918

[bib44] Schmoller, K.M., M.C. Lanz, J. Kim, M. Koivomagi, Y. Qu, C. Tang, I.V. Kukhtevich, R. Schneider, F. Rudolf, D.F. Moreno, . 2022. Whi5 is diluted and protein synthesis does not dramatically increase in pre- Start G1. Mol. Biol. Cell. 33:lt1. 10.1091/mbc.E21-01-0029 PMC9282012 35482510

[bib45] Schmoller, K.M., J.J. Turner, M. Kõivomägi, and J.M. Skotheim. 2015. Dilution of the cell cycle inhibitor Whi5 controls budding-yeast cell size. Nature. 526:268–272. 10.1038/nature14908 PMC4600446 26390151

[bib46] Schuh, L., I. Kukhtevich, P. Bheda, M. Schulz, M. Bordukova, R. Schneider, and C. Marr. 2022. Altered expression response upon repeated gene repression in single yeast cells. PLoS Comput. Biol. 18:e1010640. 10.1371/journal.pcbi.1010640 PMC9633002 36256678

[bib47] Shabestary, K., C. Klemm, B. Carling, J. Marshall, J. Savigny, M. Storch, and R. Ledesma-Amaro. 2024. Phenotypic heterogeneity follows a growth-viability tradeoff in response to amino acid identity. Nat. Commun. 15:6515. 10.1038/s41467-024-50602-8 PMC11297284 39095345

[bib48] Skotheim, J.M., S. Di Talia, E.D. Siggia, and F.R. Cross. 2008. Positive feedback of G1 cyclins ensures coherent cell cycle entry. Nature. 454:291–296. 10.1038/nature07118 PMC2606905 18633409

[bib49] Soifer, I., and N. Barkai. 2014. Systematic identification of cell size regulators in budding yeast. Mol. Syst. Biol. 10:761. 10.15252/msb.20145345 PMC4299602 25411401

[bib50] Soifer, I., L. Robert, and A. Amir. 2016. Single-cell analysis of growth in budding yeast and bacteria reveals a common size regulation strategy. Curr. Biol. 26:356–361. 10.1016/j.cub.2015.11.067 26776734

[bib51] Sommer, R.A., J.T. DeWitt, R. Tan, and D.R. Kellogg. 2021. Growth-dependent signals drive an increase in early G1 cyclin concentration to link cell cycle entry with cell growth. Elife. 10:e64364. 10.7554/eLife.64364 PMC8592568 34713806

[bib52] Stockwell, S.R., and S.A. Rifkin. 2017. A living vector field reveals constraints on galactose network induction in yeast. Mol. Syst. Biol. 13:908. 10.15252/msb.20167323 PMC5293160 28137775

[bib53] Swaffer, M.P., J. Kim, D. Chandler-Brown, M. Langhinrichs, G.K. Marinov, W.J. Greenleaf, A. Kundaje, K.M. Schmoller, and J.M. Skotheim. 2021. Transcriptional and chromatin-based partitioning mechanisms uncouple protein scaling from cell size. Mol. Cell. 81:4861–4875.e7. 10.1016/j.molcel.2021.10.007 PMC8642314 34731644

[bib54] Taberner, F.J., I. Quilis, and J.C. Igual. 2009. Spatial regulation of the start repressor Whi5. Cell Cycle. 8:3010–3018. 19713766

[bib55] Talavera, R.A., B.E. Prichard, R.A. Sommer, R.M. Leitao, C.J. Sarabia, S. Hazir, J.A. Paulo, S.P. Gygi, and D.R. Kellogg. 2024. Cell growth and nutrient availability control the mitotic exit signaling network in budding yeast. J. Cell Biol. 223:e202305008. 10.1083/jcb.202305008 PMC11082370 38722822

[bib56] Travesa, A., T.I. Kalashnikova, R.A.M. de Bruin, S.R. Cass, C. Chahwan, D.E. Lee, N.F. Lowndes, and C. Wittenberg. 2013. Repression of G_1_/S transcription is mediated via interaction of the GTB motifs of Nrm1 and Whi5 with Swi6. Mol. Cell. Biol. 33:1476–1486. 10.1128/MCB.01333-12 PMC3624247 23382076

[bib57] Tyers, M., G. Tokiwa, R. Nash, and B. Futcher. 1992. The Cln3-Cdc28 kinase complex of S. cerevisiae is regulated by proteolysis and phosphorylation. EMBO J. 11:1773–1784. 10.1002/j.1460-2075.1992.tb05229.x PMC556635 1316273

[bib58] Virtanen, P., R. Gommers, T.E. Oliphant, M. Haberland, T. Reddy, D. Cournapeau, E. Burovski, P. Peterson, W. Weckesser, J. Bright, . 2020. SciPy 1.0: Fundamental algorithms for scientific computing in Python. Nat. Methods. 17:261–272. 10.1038/s41592-019-0686-2 PMC7056644 32015543

[bib59] Wijnen, H., and B. Futcher. 1999. Genetic analysis of the shared role of CLN3 and BCK2 at the G1-S transition in *Saccharomyces cerevisiae*. Genetics. 153:1131–1143. 10.1093/genetics/153.3.1131 PMC1460821 10545447

[bib60] Xiao, J., J.J. Turner, M. Kõivomägi, and J.M. Skotheim. 2024. Whi5 hypo- and hyper-phosphorylation dynamics control cell-cycle entry and progression. Curr. Biol. 34:2434–2447.e5. 10.1016/j.cub.2024.04.052 PMC11247822 38749424

[bib61] Zatulovskiy, E., S. Zhang, D.F. Berenson, B.R. Topacio, and J.M. Skotheim. 2020. Cell growth dilutes the cell cycle inhibitor Rb to trigger cell division. Science. 369:466–471. 10.1126/science.aaz6213 PMC7489475 32703881

[bib62] Zhang, S., L.F. Valenzuela, E. Zatulovskiy, L. Mangiante, C. Curtis, and J.M. Skotheim. 2024. The G_1_ -S transition is promoted by Rb degradation via the E3 ligase UBR5. Sci. Adv. 10:eadq6858. 10.1126/sciadv.adq6858 PMC11498223 39441926

