# Supplementary material for: Single-cell imaging reveals a key role of Bck2 in yeast cell size adaptation to nutrient challenges
Source: J Cell Biol. 2026 Oct 9;225(12):e202410020. doi: 10.1083/jcb.202410020 (PMC13651637; doi:10.1083/jcb.202410020)
Supplement: Supplementary file 1 [file jcb_202410020_tables1.docx]

| **Name** | **Genotype** | **Source** | **Figures used in** |
| --- | --- | --- | --- |
| MMY116-2C | *MATα ADE2* | Lab Stock | “WT” in 1, 2, 3, 4, 5, 7, S1, S4 & S5 |
| YCY001-1 | *MATα ADE2 bck2∆::CglaTRP1* | This study | “*bck2∆”* in 1, 4, 5, 7, S1, S4 & S5 |
| YCY002-1 | *MATα ADE2 ccr4∆::CglaTRP1* | This study | “*ccr4∆*” in 1, S1 |
| YCY004-5 | *MATα ADE2 whi5∆::KlacURA3* | This study | “*whi5∆”* in 1, 4, 5 & 7 |
| YCY005-1 | *MATα ADE2 bck2∆::CglaTRP1 whi5∆::KlacURA3* | This study | “*whi5∆bck2∆”* in 1, 2, 4, 5, 7, S4 & S5 |
| YCY006-1 | *MATα ADE2 ccr4∆::CglaTRP1 whi5∆::KlacURA3* | This study | “*whi5∆ccr4∆”* in 1 & S1 |
| YCY024-1 | *MATα ADE2 ura3::Act1promoter-WHI5WIQ-mCitrine-URA3*  *htb2::HTB2-mScarlet-I-natMX6* | This study | “WT” in 6 |
| YCY030-2 | *MATα ADE2 ura3::Act1promoter-WHI5WIQ-mCitrine-URA3*  *htb2::HTB2-mScarlet-I-natMX6 whi5∆::CglaTRP1* | This study | “*whi5∆”* in 6 |
| YCY037-1 | *MATα ADE2 ura3::Act1promoter-WHI5WIQ-mCitrine-URA3*  *htb2::HTB2-mScarlet-I-natMX6 bck2∆::CglaLEU2* | This study | “*bck2∆”* in 6 |
| YCY038-1 | *MATα ADE2 ura3::Act1promoter-WHI5WIQ-mCitrine-URA3*  *htb2::HTB2-mScarlet-I-natMX6 whi5∆::CglaTRP1 bck2∆::CglaLEU2* | This study | “*whi5∆bck2∆”* in 6 |
| YCY021-2 | *MATα ADE2 cln3∆::CglaLEU2* | This study | “*cln3∆”* in 7 & S6 |
| YCY022-2 | *MATα ADE2 cln3∆::CglaLEU2 whi5∆::KlacURA3* | This study | “*whi5∆cln3∆”* in 7 & S4 |
| YCY023-1 | *MATα ADE2 cln3∆::CglaLEU2 whi5∆::KlacURA3 bck2∆::CglaTRP1* | This study | “*whi5∆bck2∆cln3∆”* in 7 & S4 |
| KSY015-5 | *MATα ADE2 cln3::LEU2-Met25promoter-CLN3* | Lab Stock | “*METpr-CLN3*” in 7D-F |
| YCY041-2 | *MATα ADE2 cln3::LEU2- Met25promoter-CLN3 bck2∆::CglaTRP1* | This study | “*METpr-CLN3* *bck2∆*” in 7D-F |
| YCY044-2 | *MATα ADE2* *cln3:: LEU2- Met25promoter-CLN3 bck2∆::CglaTRP1 whi5∆::KlacURA3* | This study | “*METpr-*CLN3 *whi5∆bck2∆*” in 7D-F |
| KSY041-2 | *MATα ADE2 cln3:: LEU2-Met25promoter-CLN3-11A-natMX6* | Lab Stock | “*METpr-CLN3-11A*” in 7D-F |
| YCY042-1 | *MATα* *ADE2 cln3:: LEU2- Met25promoter-CLN3-11A-natMX6 bck2∆::CglaTRP1* | This study | “*METpr-CLN3-11A* *bck2∆*” in 7D-F |
| YCY045-3 | *MATα* *ADE2 cln3::LEU2- Met25promoter-CLN3-11A- natMX6 bck2∆::CglaTRP1 whi5∆::KlacURA3* | This study | “*METpr-CLN3-11A whi5∆bck2∆*” in 7D-F |

**Supplemental table 1:** Table describing the strains used in this study. All the strains used are haploid derivatives of W303, constructed using standard methods and verified by sequencing.
